# Atomically resolved electronic properties in single layer graphene on α-Al_2_O_3_ (0001) by chemical vapor deposition

**DOI:** 10.1038/s41598-022-22889-4

**Published:** 2022-11-05

**Authors:** Henrik Wördenweber, Silvia Karthäuser, Annika Grundmann, Zhaodong Wang, Stephan Aussen, Holger Kalisch, Andrei Vescan, Michael Heuken, Rainer Waser, Susanne Hoffmann-Eifert

**Affiliations:** 1 Peter Grünberg Institute 7, Forschungszentrum Jülich GmbH and JARA-FIT, 52425 Jülich, Germany; 2 JARA-Institute Energy-Efficient Information Technology (Green IT & PGI-10), Forschungszentrum Jülich GmbH, 52425 Jülich, Germany; 3 Institute of Materials in Electrical Engineering and Information Technology II, RWTH Aachen University, 52074 Aachen, Germany; 4 Compound Semiconductor Technology, RWTH Aachen University, 52074 Aachen, Germany; 5 AIXTRON SE, 52134 Herzogenrath, Germany; 6 RWTH Aachen University, 52066 Aachen, Germany

**Keywords:** Two-dimensional materials, Electronic devices, Graphene, Electronic properties and materials

## Abstract

Metal-free chemical vapor deposition (CVD) of single-layer graphene (SLG) on c-plane sapphire has recently been demonstrated for wafer diameters of up to 300 mm, and the high quality of the SLG layers is generally characterized by integral methods. By applying a comprehensive analysis approach, distinct interactions at the graphene-sapphire interface and local variations caused by the substrate topography are revealed. Regions near the sapphire step edges show tiny wrinkles with a height of about 0.2 nm, framed by delaminated graphene as identified by the typical Dirac cone of free graphene. In contrast, adsorption of CVD SLG on the hydroxyl-terminated α-Al_2_O_3_ (0001) terraces results in a superstructure with a periodicity of (2.66 ± 0.03) nm. Weak hydrogen bonds formed between the hydroxylated sapphire surface and the π-electron system of SLG result in a clean interface. The charge injection induces a band gap in the adsorbed graphene layer of about (73 ± 3) meV at the Dirac point. The good agreement with the predictions of a theoretical analysis underlines the potential of this hybrid system for emerging electronic applications.

## Introduction

Two-dimensional materials (2DMs) are considered as prime candidates for significantly extending the functionality of silicon chips, referred to as ‘CMOS + X’. Co-integration of 2DMs with silicon technology raises the prospect of substantial performance and functional gains in areas like ‘More than Moore’, photonic integrated circuits, neuromorphic computing, and quantum technologies^1^. The excellent structural, thermal, and chemical stability combined with mechanical flexibility and electrical robustness may be of particular interest for memristive devices, which are considered key components for next-generation edge computing^2–6^. Recently, Wang et al. demonstrated a graphene/MoS_2−x_O_x_/graphene device that exhibits excellent resistive switching performance with an endurance of up to 10^7^ at an operation temperature of 340 °C^7^. However, further research is needed for a deeper understanding of the role of interfacial properties and defects, especially, those formed during growth and exfoliation^8^. To fully exploit the exceptional properties of 2DMs for new neuromorphic computing concepts, a scalable process compatible with semiconductor technology is needed to obtain high-quality material on technologically relevant wafer sizes^9^.

Chemical vapor deposition (CVD) has proven to be a reliable, reproducible, and technologically viable synthesis route to wafer-scale SLG films characterised by good crystallinity, low impurity densities and full compatibility with large-scale back-end-of-line (BEOL) integration. Large-area SLG were initially fabricated by CVD on Cu, which serves as a catalyst for the decomposition of hydrocarbon sources^10–13^. However, impurities resulting from imperfect removal of metal catalysts and the PMMA (poly(methyl 2-methylpropenoate)), which is required for the transfer processes, hinder the use of this material for high-volume production while meeting semiconductor standards^14,15^. Therefore, current research interest is focused on direct graphene growth on insulating substrates compatible with silicon technology such as α-Al_2_O_3_ (0001)^16–20^. This standard substrate in compound semiconductor technology guarantees large-volume availability of large-diameter wafers with the required quality at reasonable cost^21^. Moreover, the lattice constant of the c-plane of sapphire, 0.476 nm, is almost twice that of graphene (2 × 0.247 nm)^22^. Recent studies have demonstrated the suitability of c-plane sapphire wafers for direct CVD of high-quality SLG^17,23,24^ and the up-scaling to 150 mm diameter substrates using a production scale reactor (AIXTRON CCS 2D)^25^. Since the first reports of direct growth of SLG on sapphire, the alignment of the SLG on different sapphire surfaces has been the subject of several investigations. Entani et al. and Dou et al. reported a strong interfacial interaction between graphene and α-Al_2_O_3_ (0001) dominated by electrostatic forces in the graphene π-system and unsaturated electrons of the oxygen layer of the α-Al_2_O_3_ (0001) surface forming a C–O–Al interfacial bond^26,27^. In contrast, Saito et al. and Ueda et al. found that graphene growth on c-plane sapphire starts from etch pits formed during the CVD process. The Al-rich surface within the pits plays a central role in the catalytic activity for SLG growth^28,29^. This assertion is also supported by the work of Mishra et al. and Chen et al., who obtained high quality CVD SLG for α-Al_2_O_3_ (0001) treated in a hydrogen atmosphere prior to the graphene deposition at high temperatures of 1180 °C and 1400 °C, respectively^20,25^. Room temperature carrier mobility values of over 2000 cm^2^/Vs and 6000 cm^2^/Vs were reported. In contrast to SLG grown on untreated wafers, these films exhibited a lower density of ridges, well defined atomic terraces, and improved crystalline quality with an averaged full width at half maximum (FWHM) of the 2D Raman mode of about 30 cm^-1^ to 35 cm^−1^. The low D/G and high 2D/G intensity ratios of about 0.15 and well above 2, respectively, indicate low defect density and carrier concentration in the lower 10^12^ cm^−2^ range^25^. However, the increasing interest in using CVD SLG on hydrated α-Al_2_O_3_ (0001) to realize nanoscale electronic devices for next-generation electronics, optoelectronics, quantum and neuromorphic computing, requires a more detailed physicochemical understanding of the electronic properties of the SLG/sapphire system down to the atomic scale^30–32^. In particular, the potential energy surface of the SLG on sapphire stack affects both the interface with subsequently deposited layers of sp^2^-hybridized 2DMs such as h-BN and transition metal dichalcogenides and the characteristic properties of functional devices such as reliability, endurance and retention.

Therefore, this study focuses on the analysis of local electronic transport properties of CVD SLG on α-Al_2_O_3_ (0001) provided by AIXTRON SE. Raman spectroscopy and Raman mapping were combined with scanning electron microscopy (SEM), conductive atomic force microscopy (c-AFM) and Hall measurements in van der Pauw geometry realized by vapor deposited gold contacts. Surface chemical characterization was performed by X-ray photoelectron spectroscopy (XPS). Atomic level electronic properties were analyzed by scanning tunneling microscopy (STM) and spectroscopy (STS). The combination of micro- and nanoscale analyses provided a deeper understanding of local variations in the SLG/sapphire interactions and the weak electrostatic bonding that controls the electronic properties of the system. A comparison with previously published results from advanced-principles calculations^33^ complements the study.

## Experiments

### Sample information

SLG was deposited on c-plane sapphire with an offcut of 0.2° (SLG/ α-Al_2_O_3_ (0001)) in an AIXTRON CCS 2D system in 19 × 2″ configuration. The deposition process was essentially similar to the one described in Ref.^25^. In a pre-bake step, the sapphire substrates were etched in H_2_ atmosphere at 1400 °C for 10 min. This was followed by graphene deposition at 1460 °C for 500 s using a CH_4_/H_2_ mixture in N_2_ atmosphere at 700 mbar. The SLG/α-Al_2_O_3_ (0001) wafers were cleaved into smaller pieces and subsequently, stored in vacuum or under inert gas for further analysis. The transport of the samples or cleaved pieces between the different measurement or storage facilities was done in closed boxes. No further treatment was performed prior to measurement.

### Methods

µ-Raman spectroscopy in mapping mode was performed at room temperature utilizing the confocal Raman microscope XploRA™ Plus of Horiba equipped with a solid-state laser with a wavelength of 532 nm at 8 mW. The laser line was focused on the sample by a 100 × microscope objective lens resulting in a spot size of about 0.5 µm. The collected light was scattered through a grating with 1200 grooves/mm. Mappings were performed with an exposure time of 2 s, an accumulation time of 2 s and a step size of 0.4 µm. The data were processed using LabSpec 6 spectroscopic suite software from HORIBA. First, a baseline subtraction is performed, then the peak position is determined by the peak maximum. Intensity ratios are calculated from the maximum intensity values and FWHM-values are determined at half maximum intensity. Comparison with the Lorentzian fit of the peaks provides comparable values considering the error ranges. XPS measurements were performed with a VersaProbe 5000 from Physical Electronics. Monochromatic Al K_α_ radiation with an excitation energy of 1486.6 eV and a beam diameter of 100 µm was used. The binding energy scales of the XPS spectra were calibrated to the C 1s peak and to the Al 2p peak at 285 eV and 74.1 eV, respectively. Survey scans and core level spectra of Al 2p, O 1s, and C 1s were recorded in the low-power mode at 25 W with an X-ray spot diameter of about 100 µm. To compensate for charging effects electron neutralization was performed with a neutralizer emission current of 20 µA and a neutralizer bias of 1.37 eV. Survey scans were performed with 187 eV pass energy. The high resolution scans measured at a take-off angle of 45° and a pass energy of 11.75 eV were used to perform the quantitative analysis. The spectra were analyzed with CasaXPS software, Version 2.3.23PR1.0. For the XPS core level analyses, a Shirley background profile was subtracted from all core level spectra. The C sp^2^ peak was fitted in CasaXPS with an asymmetric peak shape defined as A (0.4, 0.38, 20) GL (20) while all other components were fitted by symmetric peak shapes as GL (30). Samples were stored in nitrogen to minimize atmospheric exposure. Carrier type, mobility and sheet density were obtained from Hall effect measurements at room temperature and a magnetic field of 0.2 T using a LakeShore 8404 AC/DC Hall effect measurement system. The SLG/sapphire samples were cleaved into 10 mm × 10 mm pieces and contacted by Pt metal pads in conventional van der Pauw geometry. SEM images were acquired using a Hitachi SU8000 operating at an acceleration voltage of 0.5 kV and a chamber pressure around 10^–7^ mbar. The SLG was grounded during these SEM measurements. For c-AFM and STM measurements a smaller piece of the sample (max. 10 × 10 mm^2^) was placed on an Omicron sample holder. Two small metal sheets were used to fix the sample and make a conductive contact to the SLG which served as a back contact. AFM and c-AFM measurements were executed under ambient conditions. AFM and c-AFM measurements were performed in tapping and contact modes in a Cypher AFM (Asylum Research) with commercial AFM probes (Nanosensors™). STM and STS measurements were performed using a low-temperature (LT) STM from CreaTec Fischer. The STM was operated under ultra-high vacuum (UHV) with a chamber pressure below 10^–10^ mbar at 4.2 K using custom-made electrochemically etched W tips. Unless otherwise specified, the following systems’ settings were used: STM measurements were performed with an applied bias voltage of 2.3 V and a set-point current of 0.23 nA in constant-current mode. STS measurements were made in the range of + 1.0 to − 1.0 V with feedback loop turned off. The differential tunnel current was determined using an internal lock-in amplifier operating at 473 Hz and an amplitude of 80 mV. The STM images were plane-corrected using the SPIP™ analytical software of Image Metrology A/S, with an optional noise filter.

## Results and discussion

### Spectroscopic characterization of SLG on H_2_-etched sapphire

**Figure 1:**
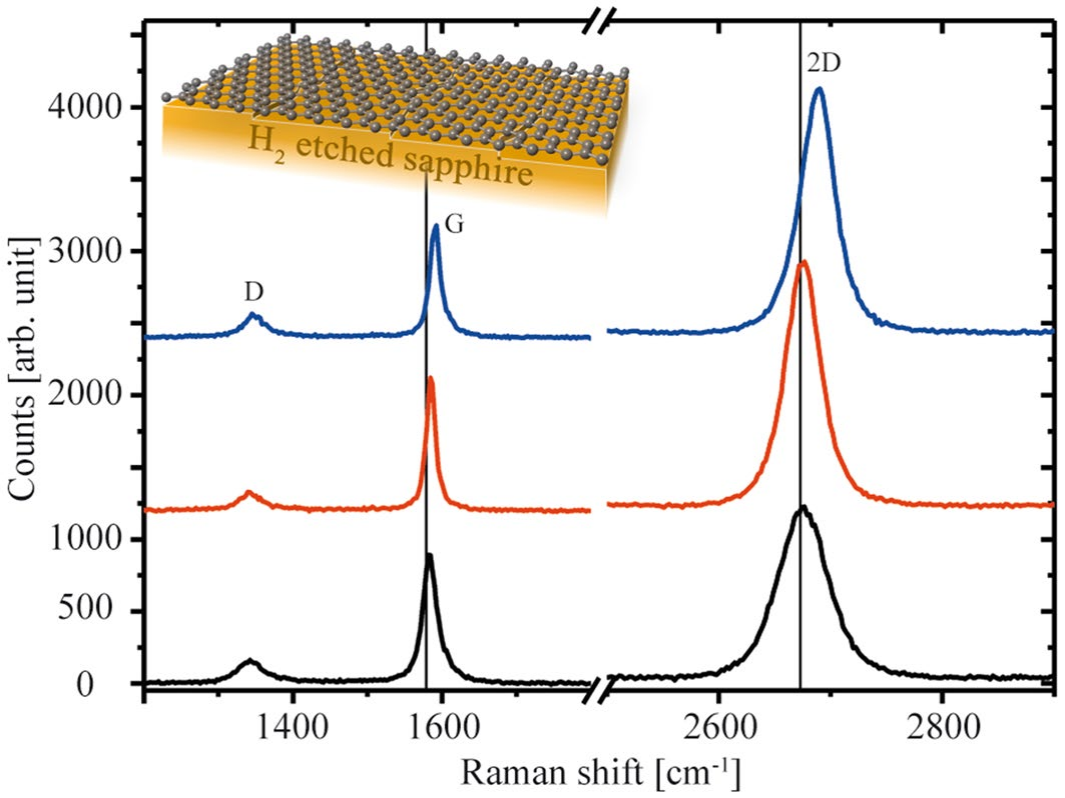
Raman measurements of SLG on H_2_-etched sapphire. The three characteristic spectra are related to different local positions arbitrarily selected from Raman mappings. The characteristic values obtained from each spectrum are summarized in Table 1. The sample structure is sketched in the inset (Adobe Photoshop Version 13.0, Adobe Illustrator CS6 Version 16.0; both: http://www.adobe.com).

The sample structure is outlined in the inset of Fig. 1. Representative Raman spectroscopy data were extracted from Raman mapping measurements in which different areas of 10 µm × 10 µm in size were scanned with a spot size on the surface of about 500 nm and a distance between the measurements of 400 nm. Raman mappings and additional spectra are shown in Fig. S1 of the Supplementary Information. All spectra in Fig. 1 contain the main peaks of graphene: D, G, and 2D^34,35^. Characteristic values such as the positions of the G and the 2D peaks, Δω_G_ and Δω_2D_, respectively, the full width at half maximum (FWHM_G_ and FWHM_2D_), and the intensity ratios *I*_2D_/*I*_G_ and *I*_D_/*I*_G_ are shown in Table 1. The curve fitting of the 2D peaks show that they have a simple Lorentz shape. The intensity ratio *I*_2D_/*I*_G_ can be used to qualitatively estimate whether the analysed graphene film is SLG or multilayer graphene. It is noticeable that the intensity ratio depends on the Raman setup, especially on the laser wave length and the chosen grating^35,36^. Peak positions and FWHM depend on the number of layers, but also on the density of defects and on strain effects^37^.

**Table 1:** Characteristics of the Raman spectra shown in Fig. 1.

Spectrum	*I*_D_/*I*_G_	*I*_2D_/*I*_G_	Δω_G_ (cm^−1^)	Δω_2D_ (cm^−1^)	FWHM_G_ (cm^−1^)	FWHM_2D_ (cm^−1^)
Blue	0.25 ± 0.06	2.4 ± 0.2	1589 ± 2	2691 ± 2	19 ± 2	37 ± 2
Red	0.19 ± 0.02	2.2 ± 0.1	1584 ± 1	2674 ± 1	16 ± 1	35 ± 3
Black	0.22 ± 0.08	1.5 ± 0.4	1582 ± 1	2672 ± 4	22 ± 4	53 ± 4

Moreover, the D/G intensity ratio is reciprocally related to the defect concentration of SLG and also correlates with the grain size^38^. *I*_D_/*I*_G_ values in the range of 0.2 indicate moderate defect density in our samples^23,39^. The thin vertical lines in Fig. 1 show the positions of the G and 2D peaks for free-standing graphene at 1579 cm^−1^ and 2673 cm^−1^, respectively^40^. A slight shift of the peaks to higher values, corresponding to a blue shift, indicates minor compressive strain, probably induced during cooling from the deposition temperature^37^. Since coefficients of thermal expansion of graphene and sapphire are different over the entire temperature range - negative for graphene and positive for sapphire - a lateral compressive stress is induced in the graphene layer during cooling^41–43^.

Although the lateral resolution of the mapping is still moderate at less than 500 nm, there is a clear local inhomogeneity in the quality of the graphene layer, in agreement with the report of Neumann et al.^37^. Figure 1 shows example spectra overing the range of possible variations, and the corresponding characteristic values are given in Table 1. Three main types of Raman spectra are obtained: (1) the black curve shows the presence of multilayer graphene with an intensity ratio of *I*_2D_/*I*_G_ < 2. Moreover, the linewidth of the 2D peak is very broad in the 53 cm^−1^ region, but the peak positions almost orrespond to the free-standing graphene. (2) The red spectrum meets the SLG criteria considering *I*_2D_/*I*_G_ > 2 and shows the smallest peak widths of all curves with FWHM values of about 16 cm^−1^ and 35 cm^−1^ for the G and 2D peaks, respectively. (3) The blue spectrum also meets the SLG criteria, but shows a significant blue shift in the G and 2 -peak positions compared to free-standing graphene, which can be attributed to a compressive stress effect. The *I*_D_/*I*_G_ ratio of the blue curve is slightly increased compared to the red curve consistent with an influence of strain in addition to defects from the growth process. Compared to the results of Tsoukleri et al.^44^, who subjected a graphene monolayer to tensile and compressive strain, the obtained peak shift of the blue curve could be attributed to a local compressive strain of about 0.3%. Despite the limited lateral resolution of the Raman measurements, the mapping gives evidence of local differences in the quality of the graphene layer, predominantly SLG, grown on the sapphire substrate by CVD. Van der Pauw measurements performed on samples of about 10 mm × 10 mm in size show an overall slight p-type conduction of the SLG with a mobility at room temperature of (1500 ± 100) cm^2^/Vs and a sheet carrier concentration of about 2.22 × 10^12^ cm^−2^, which are in a reasonable range compared to the range in the literature, from outstanding values of about 6000 cm^2^/Vs^20^ to values commonly reported for CVD graphene on dielectric substrates, typically below 1000 cm^2^/Vs^45^. It appears that the H_2_-etched α-Al_2_O_3_ (0001) surface has a smaller effect on the electronic properties of SLG than other dielectric substrates.

**Figure 2:**
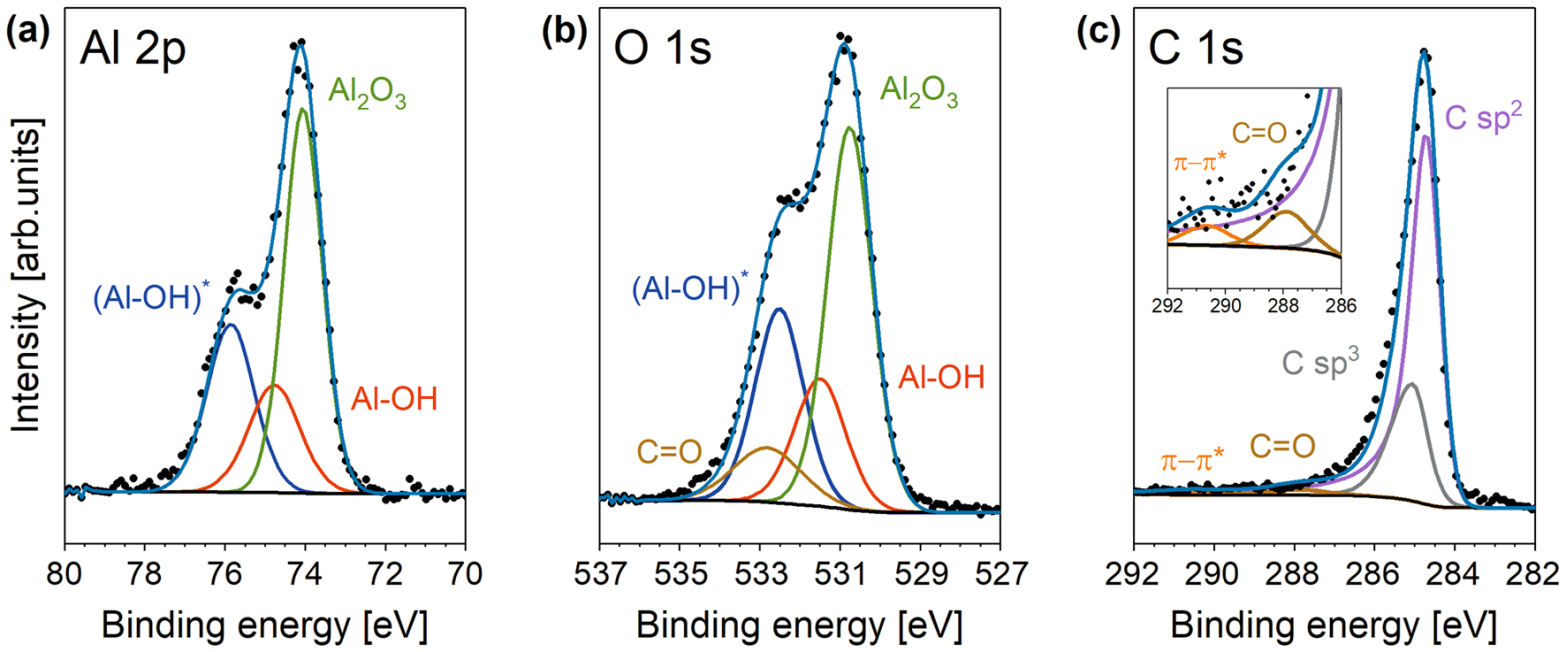
Core-level spectra of SLG on H_2_-etched sapphire for a take-off angle of 45° and a pass energy of 11.75 eV: (**a**,**b**) show the Al 2p and O 1s spectra, which are essentially related to the sapphire surface; (**c**) shows the C 1s spectrum originating from the graphene layer and adsorbed carbon species; inset shows magnification. The black dots show the raw data, the steel blue lines show the envelopes of the fitted components, and the black lines show the subtracted Shirley backgrounds. The following components are used for the fit: (Al–OH)* in blue, Al–OH in red, Al_2_O_3_ in green, C=O in brown, π–π* in orange, C sp^3^ in grey and C sp^2^ in violet. Details are summarized in Table 2.

In order to characterize the graphene surface and the graphene/substrate interface chemically in more detail, XPS measurements were performed. Survey scans and core level spectra of Al 2p, O 1s and C 1s were recorded for an untreated sapphire substrate, for an α-Al_2_O_3_ (0001) sample after prebaking in hydrogen at 1400 °C for 10 min, and for the SLG/sapphire sample. For the analysis of the untreated and the prebaked sapphire substrates, the energy scale was calibrated using the C 1s signal of adsorbed carbon at 285.0 eV. Due to the overlap of the C 1s signals from adsorbed carbon species and the graphene layer for the SLG/sapphire samples, the energy scale was calibrated with respect to the Al 2p peak of Al_2_O_3_ at a binding energy of 74.1 eV^46^. Figure S2a summarizes the survey scans of the original α-Al_2_O_3_ (0001), the sapphire surface prebaked with H_2_, and the SLG/sapphire samples as black, blue, and red lines, respectively. For all scans, only peaks attributable to oxygen, carbon and aluminium are observed. Other peaks such as those from impurities, are not seen. The Al 2p, O 1s, and C 1s core level spectra of the sapphire substrate in the untretaed and H_2_-prebaked state are shown in Fig. S2b–g. Figure 2a–c show the respective core level spectra of the SLG/sapphire sample. The spectra were analyzed with CasaXPS software, Version 2.3.23PR1.0. A Shirley background profile (black line) was subtracted from all core level spectra. Components were fitted in CasaXPS with symmetric peak shapes as GL (30), except for the C sp^2^ peak which was fitted with an asymmetric peak shape as A (0.4, 0.38, 20) GL(20)^47^. The peak energies and widths (FWHM) of the chemical components fitted to the spectra are listed in Table S2 of the Supplementary Information and in Table 2 for the SLG/sapphire sample. For the pristine sapphire substrate (Fig. S2b-d) the core level spectra can be fitted throughout by adsorbed carbon, resulting in  a C 1s peak at 285.0 eV and a C=O component in the O 1s spectrum at 532.3 eV. The Al 2p peak at 74.1 eV and the O 1s peak at 530.8 eV are attributed to Al_2_O_3_^48^. After prebaking at 1400 °C in H_2_ additional peaks appear in the Al 2p and O 1s spectra at about 74.8 eV and 531.5 eV, respectively (Fig. S2e,f), which are attributed to Al–OH species^48^. The dependence of their intensity on the take-off angle confirms a near-surface position.

**Table 2:** Binding energies and full widths at half maximum (FWHM) of the core levels of Al 2p, O 1s, and C 1s of the SLG/α-Al_2_O_3_ system after calibration of Al 2p (Al_2_O_3_) to 74.1 eV.

Core level	Al 2p			O 1s				C 1s			
Compo-nent	Al_2_O_3_	Al–OH	(Al–OH)*	Al_2_O_3_	Al–OH	(Al–OH)*	C=O	C sp^2^	C sp^3^	C=O	π–π*
Binding energy (eV)	74.1	74.8	75.9	530.8	531.5	532.5	532.8	284.7	285.0	287.9	290.8
FWHM (eV)	1.1	1.5	1.4	1.3	1.5	1.4	2.0	0.8	0.9	1.5	2.1

The spectra of Al 2p and O 1s core levels are also considered as indicators of the chemical state of the H_2_-etched sapphire surface after coating with SLG. Figure 2a,b show the presence of Al_2_O_3_ groups and Al–OH groups at the interface identified by peaks at binding energies observed for the untreated and the H_2_-prebaked sapphire surface. The Al 2p and the O 1s core level spectra give the same ratio of the two peak intensities [Al–OH]/[Al_2_O_3_] of about 0.38 at 45° take-off angle. However, additional peaks with higher binding energies compared to Al–OH must be introduced to fit the measured spectra. The binding energies of this additional component (Al–OH)* are determined to be 75.9 eV and 532.5 eV for Al 2p and O 1s, respectively. Again, the ratio of the peak intensities [(Al–OH)*]/[Al_2_O_3_] for the two core level spectra is constant with a value of about 0.55 at 45° take-off angle. The appearance of two interface components, Al–OH and (Al–OH)*, with different binding energies could indicate local inhomogeneities in the SLG/sapphire interaction. The increased binding energy of the (Al–OH)* component ompared to the literature values for Al–OH indicates the formation of hydrogen bonds. The C=O signal is strongest at an analyser angle of 63°, indicating surface species. The C 1s core level spectrum in Fig. 2c is composed of C sp^3^ and C sp^2^ signals as the major species. The C sp^2^ species and the clear signature of the π–π* (shake-up) peak at a binding energy of about 290.5 eV (see inset in Fig. 2c) demonstrate the existence of graphene on the α-Al_2_O_3_ surface^49^. The determination of the C sp^3^ carbon component at a slightly higher binding energy, about 285.0 eV, is a result of the coupling of SLG with neighboring functional groups and an indication for the interaction with the H_2_-etched sapphire^50,51^. The [C sp^3^]/[C sp^2^] ratio is nearly constant at about (0.44 ± 0.01), regardless of the analyzer angle.

### SLG morphology

**Figure 3:**
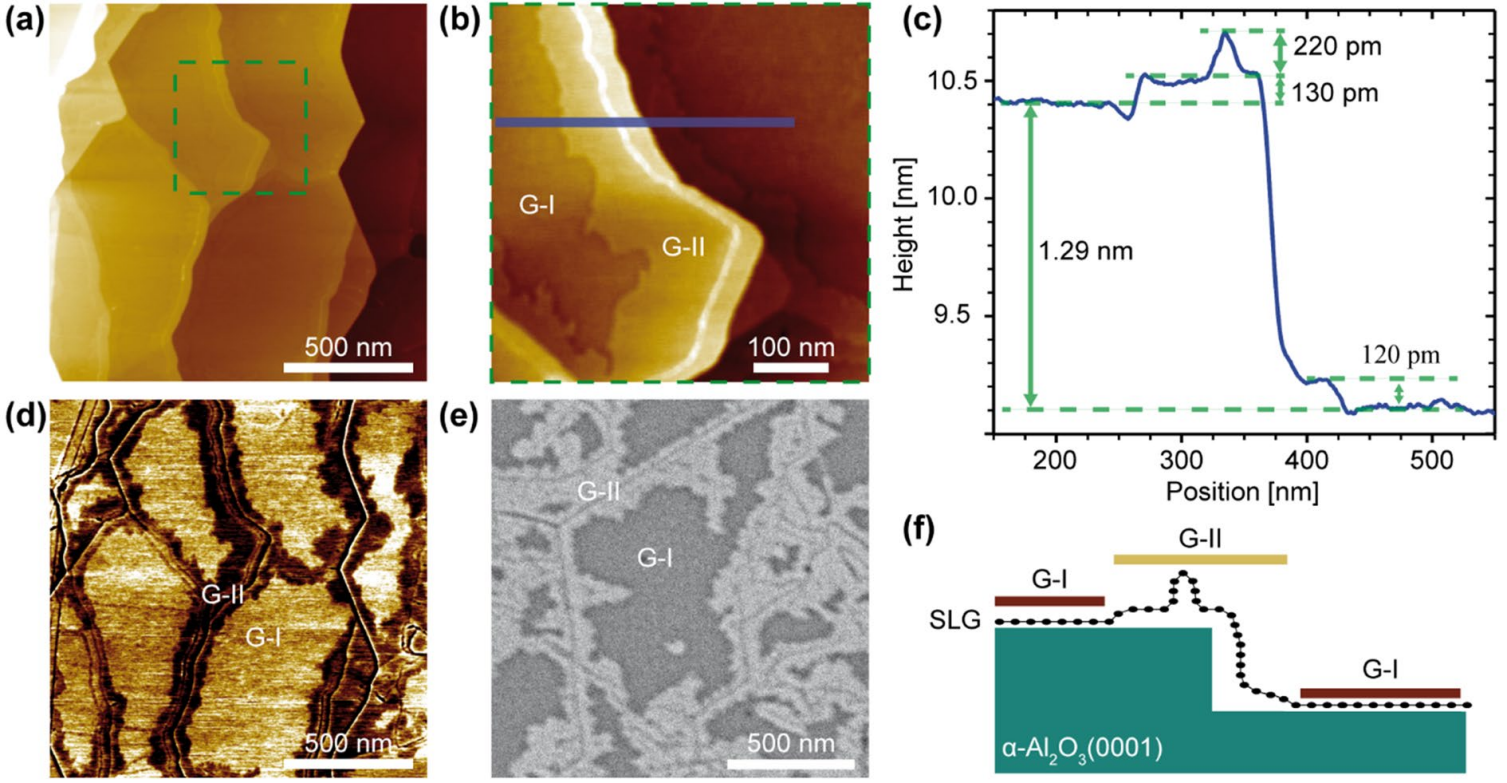
AFM and SEM measurements of SLG on α-Al_2_O_3_ (0001). (**a**) Large area AFM image showing the edges of the sapphire steps. (**b**) Higher resolution image of the area marked in (**a**) (green square) showing an α-Al_2_O_3_ (0001) step and wrinkles near the step edges. (**c**) Cross-section averaged over several lines along the blue bar in (**b**) showing a sapphire step edge of about 1.29 nm. Near the step edge, the SLG is elevated 120 pm to 130 pm from the terrace. (**d**) Corresponding phase image to (**a**). (**e**) SEM image. (**f**) Schematic of delamination near the step edges of sapphire. Two SLG regions an be seen, one on flat sapphire terraces and the other in the vicinity of sapphire step edges; they are labeled G-I and G-II, respectively, in (**b**)–(**f**).

Large-area AFM images (Fig. 3a) show a homogeneous morphology. Only sapphire step edges and capillary wrinkles are observed (Supporting Information Fig. S3). α-Al_2_O_3_ (0001) exhibits two types of step edges, smaller ones with a step height of 0.21 nm ± 0.01 nm, characteristic of monoatomic α-Al_2_O_3_ (0001) steps (1/6 c = 0.217 nm) between two oxygen layers, and larger ones with a step height of 1.30 nm ± 0.01 nm corresponding to the height of the unit cell (c = 1.299 nm) or a multiple thereof^22,52^. A unit cell step is marked in the height profile of Fig. 3c. Remarkably, no step edges corresponding to the graphene interlayer spacing of 0.33 nm could be recorded in any of the measurements, again indicating SLG coverage over the entire wafer^53,54^. The height of the observed wrinkles ranges from 0.2 nm to 0.6 nm, and a root mean square (RMS) roughness of (38 ± 2) pm was obtained for areas of 1 µm^2^ including the step edges of sapphire.

Another feature that appears in the high-resolution AFM images is a variation in the height of the SLG surface with respect to the sapphire surface (Fig. 3b,c). It should be mentioned that the RMS values in the two areas labeled G-I or G-II are (17 ± 2) pm. The vertical distance between SLG and α-Al_2_O_3_ (0001) measured in the enter of flat sapphire terraces (see Fig. 3b), is about 0.12 nm ± 0.01 nm smaller in the darker areas (G-I) than in the lighter areas (G-II) along the sapphire step edges. The G-II region also includes the wrinkles shown schematically in Fig. 3f. Obviously, SLG detaches from the sapphire surface near the step edges and follows the surface structure on the sapphire terraces. It is also likely that the wrinkles originate from strain release and complement the delamination of the SLG film^17^. Together with the Raman data indicating locally inhomogeneous strain, a consistent picture of strain release by delamination at the step edges of α-Al_2_O_3_ (0001) emerges. The height of these step edges can be as low as 0.22 nm but can rise to more than 4.0 nm (Fig. S3b). Thus, we conclude that the structure of graphene in the G-I region, i.e., the graphene adsorbed on α-Al_2_O_3_ (0001) in the terrace region, is energetically favourable compared to the delaminated graphene/sapphire structure near the step edges (G-II). However, the latter allows the release of stresses and is necessary to enable the formation of the preferred G-I structure also on the next terraces. This issue will be further discussed together with the results from STM/STS analysis.

Further insight is provided by an AFM phase contrast image taken in tapping mode (see Fig. 3d). Here, the SLG regimes G-I and G-II also appear with different brightness, indicating different tip-graphene interactions on the flat sapphire terraces and in the regions near the sapphire step edges. Moreover, the SEM images in Fig. 3e clearly show the two different SLG regimes despite their small height difference. However, brighter regions in the SEM images indicate higher electron density^55^, suggesting that SLG regions G-II along the step edges have higher carrier density than SLG regions G-I, which are more tightly bound in the enter of the sapphire terraces. In addition, SEM analysis allowed us to image these specific morphological features at larger length scales and at multiple positions on the wafer (cf. Supporting Information Fig. S4). The effect of different conductivity was further investigated using c-AFM.

### Electrical properties of SLG

**Figure 4:**
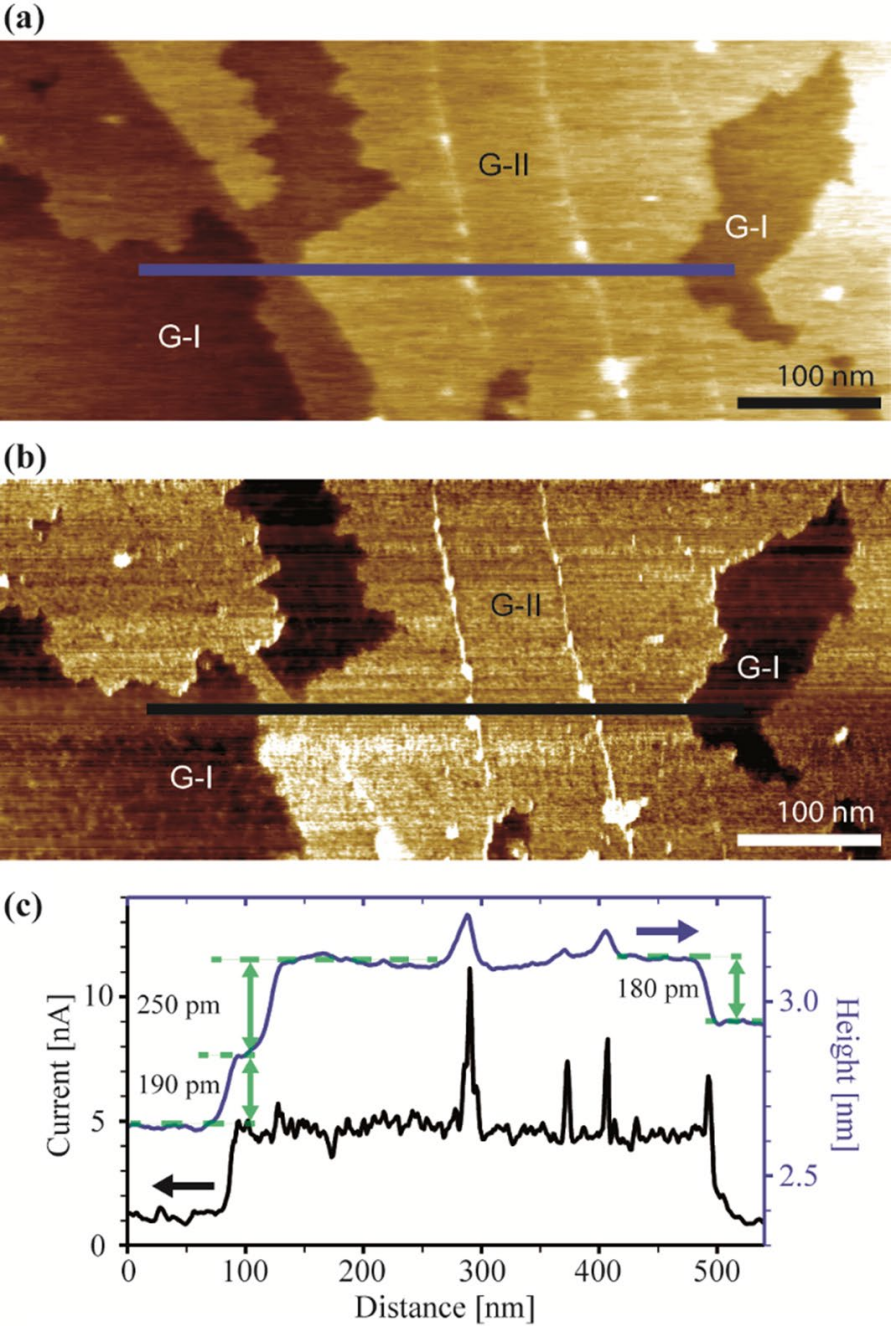
c-AFM measurements. (**a**) Topography image and (**b**) conductivity map of the same sample section. (**c**) Height (blue) and current (black) profiles along the bars indicated in (**a**) and (**b**).

The c-AFM results shown in Fig. 4 allow a direct correlation of topographic and electronic properties with a special attention to the two SLG regions, which show significantly different interactions with the sapphire surface.

Comparison of Figs. 4a and b shows that the conductivity of the SLG does not change abruptly at the sapphire step edges, but generally at a distance of 30–80 nm before or after the step edge. The line scans in Fig. 4c show that the conductivity in the detached graphene (G-II) lacated near the sapphire step edge is almost four times higher than the conductivity of the SLG attached to the sapphire terraces (G-I).

Relatively strong electrostatic interactions between SLG and the oxygen terminated α-Al_2_O_3_ (0001) surface have been reported in the literature, resulting in an interlayer spacing of 0.26 nm^26^. On the other hand, theoretical studies of SLG on the Al-terminated α-Al_2_O_3_ (0001) surface show weaker dispersion interactions and an interlayer spacing of 0.31 nm^24,26^. Importantly, a vertical spacing of 0.338 nm for the SLG/Ir(111) system and of 0.215 nm for SLG/Ni(111) indicates weak van der Waals and covalent interactions, respectively^56,57^. Strong electrostatic interactions between SLG and α-Al_2_O_3_ (0001) result from the delocalized π-electron system of SLG interacting with the dangling bonds of the α-Al_2_O_3_ (0001) surface. These dangling bonds, located at the topmost oxygen atoms, induce *p*-type doping of SLG^26^. Due to this interaction  between the layers, a decrease in electron mobility is expected. However, we found that the vertical SLG/α-Al_2_O_3_ (0001) distance is increased by 0.12 nm near the step edges of the sapphire. Assuming a minimal value of 0.26 nm for the interfacial distance on terraces, this would result in a distance of 0.38 nm (= 0.26 nm + 0.12 nm) at the step edges. This is significantly larger than the interlayer spacing of graphite (0.336 nm), indicating delamination of the SLG in this region. Delamination also explains the fourfold higher conductivity in the sapphire edge regions compared to the terrace regions of α-Al_2_O_3_, where the electron mobility is reduced due to interlayer interactions. Consequently, SLG in regime G-II is considered as ‘nearly free-standing’ graphene in the following.

### Scanning tunneling microscopy and spectroscopy

**Figure 5:**
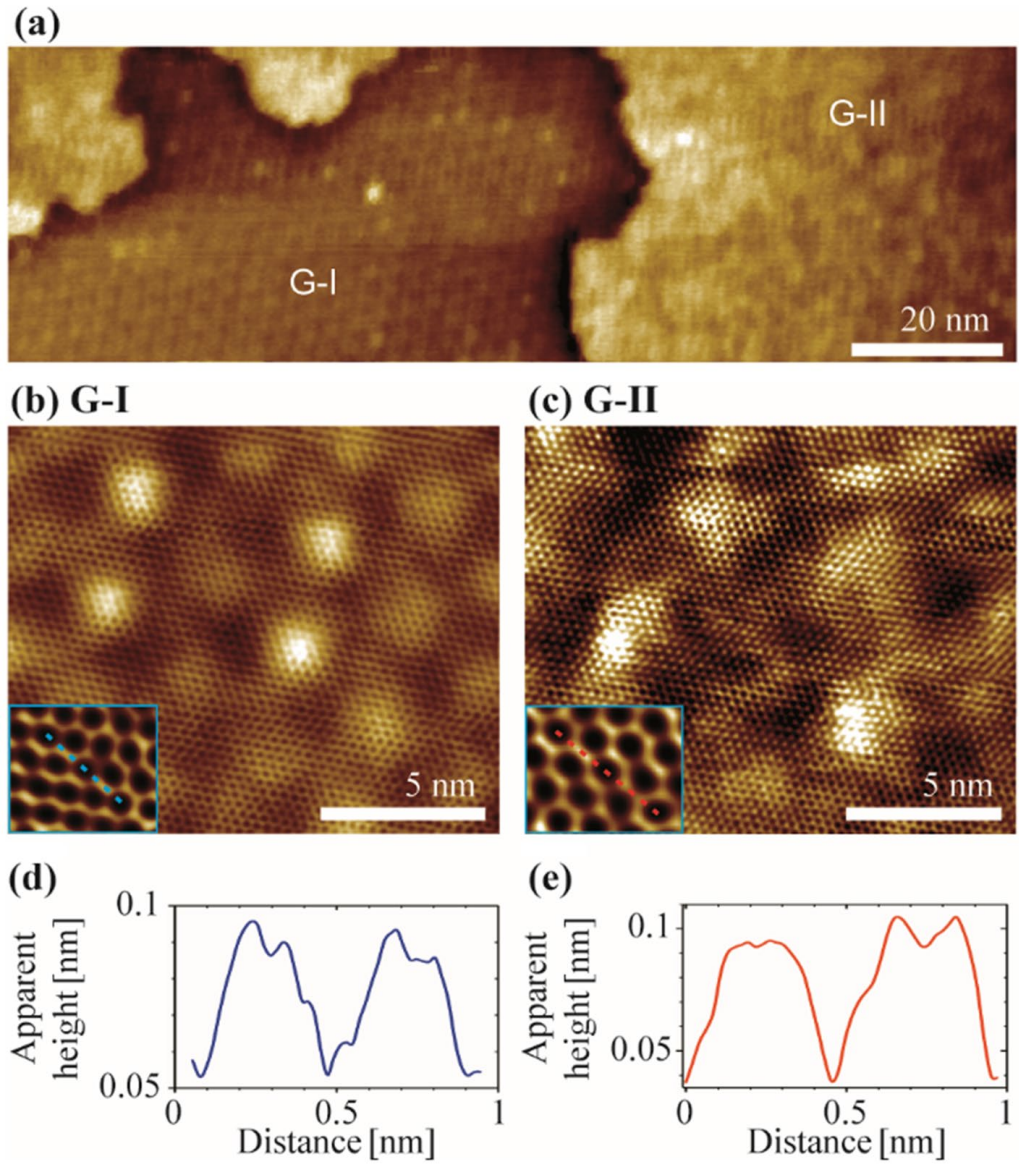
LT-UHV-STM measurements. (**a**) Overview of the two different graphene regions. (**b**) High-resolution image of G-I showing a superstructure. Inset (1.2 × 1.4) nm^2^: atomically resolved graphene structure. (**c**) G-II corresponds to the nearly free-standing SLG with random fluctuations in apparent height. Inset: atomically resolved honeycomb structure of SLG (1.2 × 1.4) nm^2^. (**d**,**e**) Apparent height profiles along the lines indicated in (**b**) and (**c**), respectively.

More detailed information about the surface structure of our samples was derived from LT-UHV-STM measurements. The right side of Fig. 5a, magnified in Fig. 5c, corresponds to SLG near a sapphire step edge (G-II), and the atomically resolved structure (inset) resembles a honeycomb pattern characteristic of a free-standing graphene layer. Here, the A and B sites of the graphene sublattices have equivalent apparent height. The contrast variations are aused by potential fluctuations originating from π–π orbital mixing enabled by ripples and step edges^58^. The resulting charge inhomogeneity is randomly distributed. The left side of Fig. 5a, enlarged in Fig. 5b, shows SLG adsorbed on a sapphire terrace (G-I), which has a periodic structure. In addition, the atomically resolved structure (inset of Fig. 5b) shows a slight triangular appearance, indicating a stronger graphene/substrate interaction in the G-I region compared to G-II. From the literature, distinct triangular appearances and﻿ ﻿﻿s﻿ymmetry﻿ breaking of the graphene lattice, indicated by unequal apparent heights of sublattices A and B, characterize strongly interacting systems such as SLG/graphite or SLG/metal interfaces^59,60^. The superstructure of SLG in the G-I regime has a period between 2.64 nm  and 2.68 nm, with the majority of buckles having an apparent height of about 45 pm and only a few buckles, shown as brighter regions in Fig. 5b, having apparent heights in the range 110 pm–150 pm^25^. The buckle structure is rotated by 27° ± 1° compared to the atomic honeycomb structure of SLG (moiré pattern analysis in Supporting Information, Fig. S5). This moiré pattern originating from a twisting angle between SLG and the α-Al_2_O_3_ (0001) surface, is onsistent with the supercells proposed for Al-terminated sapphire and, in particular, with the data reported by Mishra et al. who used a comparable deposition method for SLG^24,25^.

Thus, we find that the height difference of 120 pm and the conductance difference between G-I and G-II, which we determined by AFM and c-AFM, and which we attribute to a different interlayer coupling of graphene with the underlying α-Al_2_O_3_ (0001) in the two regions, are also visible in the STM analysis. While G-II exhibits the features of a free-standing graphene layer, a honeycomb pattern, and random contrast fluctuations, G-I shows the signs of interlayer coupling, i. e., a triangular appearance of the graphene pattern and a superstructure (Fig. 5). We measured an apparent height difference between the two graphene regions of 160 pm in our STM, which points to a roughly threefold increased conductivity of G-II compared to G-I, considering the real height differences measured in AFM. A comparable behaviour with the same change in properties was observed for graphene flakes adsorbed on graphite^61^. In this case, the height difference between the coupled graphene and the free-standing graphene was 100 pm.

**Figure 6:**
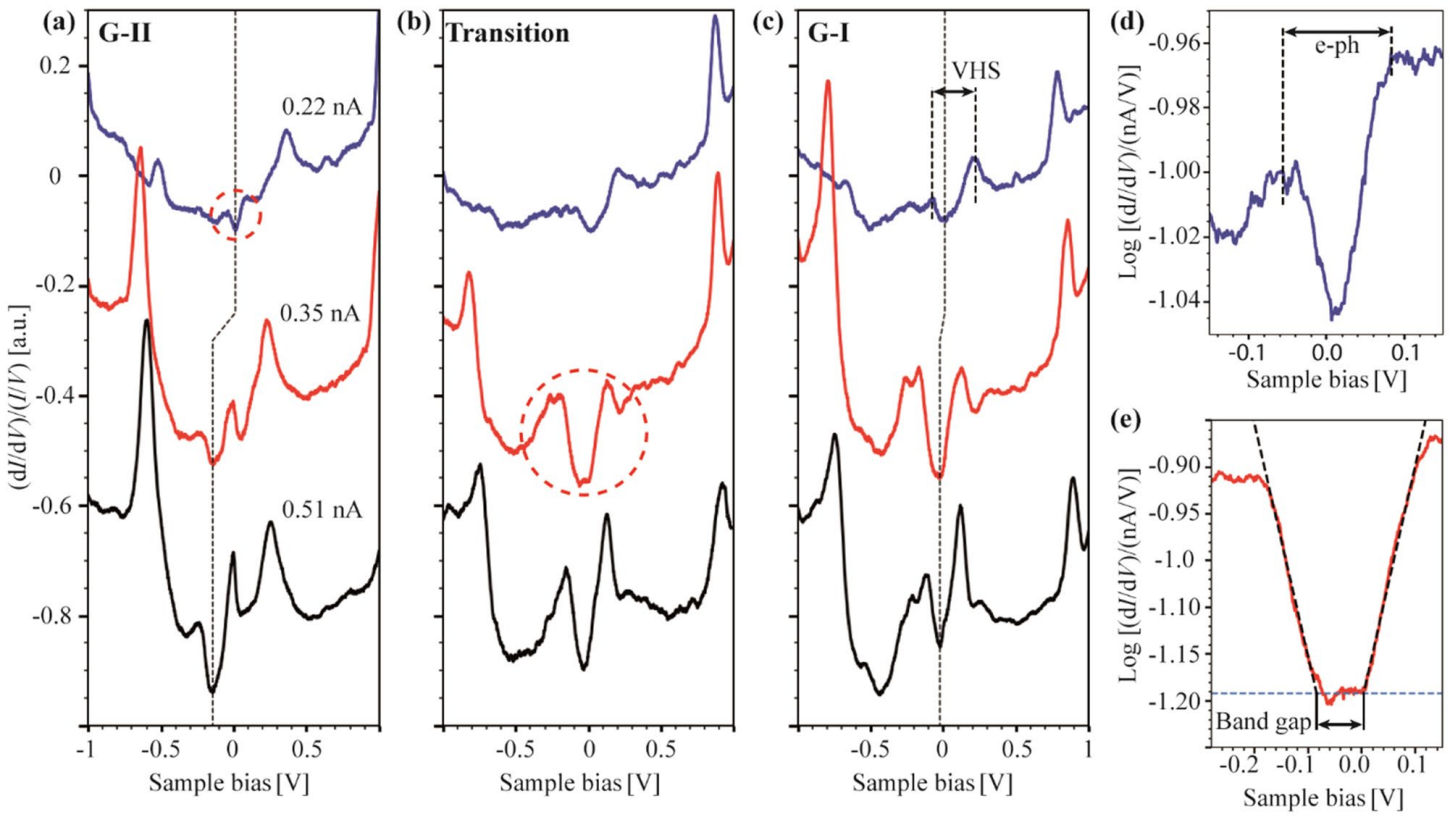
Normalized differential conductance measurements at three W tip—SLG/α-Al_2_O_3_ (0001) distances controlled by setpoint currents (0.22 nA, 0.35 nA, and 0.51 nA corresponding to the blue, red, and black curves, respectively, at *V*_bias_ = 1.0 V). (**a**) G-II region near the step edge. (**b**) Transition region between G-II and G-I. (**c**) G-I region on the sapphire terrace. A Dirac point shift as the tip is approached is indicated by the dashed vertical line. (**d**) Diagram of the Dirac cone marked in (**a**). (**e**) Plot of the substrate-induced bandgap opening marked in (**b**).

**Figure 7:**
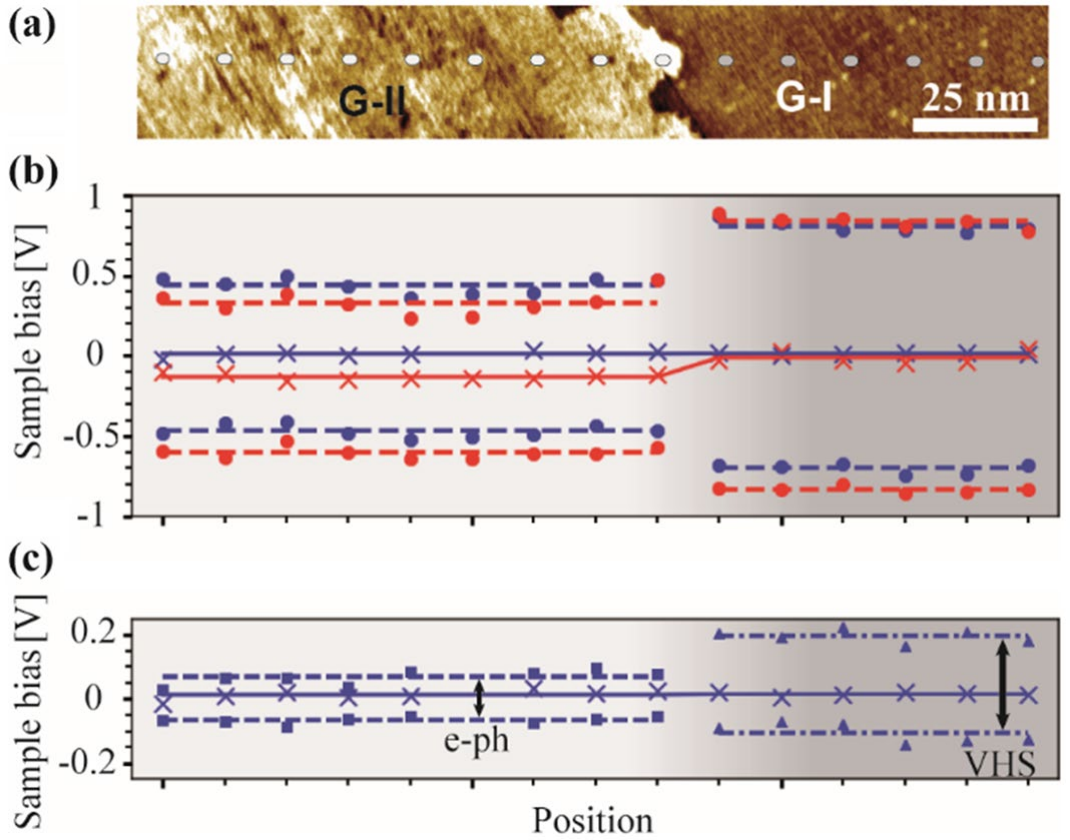
STS features along a line crossing different SLG regions from regime G-II at the left to regime G-I on the right. (**a**) STM image (*V*_bias_ = 1.0 V, *I*_set_ = 0.4 nA) of the two different SLG regions marking the 15 STS measurement positions. (**b**) Position-dependent Dirac points (cross) and main STS peaks (circle) for *I*_set_ = 0.22 nA (blue) and *I*_set_ = 0.35 nA (red). (**c**) Position-dependent Dirac points (cross), electron phonon peaks (square) and Van Hove singularity peaks (triangle) (*I*_set_ = 0.22 nA).

We clearly observed differences in the binding mechanism on the flat terraces and near the step-edges of the same sample leading to different electronic properties in the respective regions. These results are of obvious significance for the design of devices using SLG/sapphire as the bottom electrode/substrate stack. Therefore, the local electronic properties of our SLG/α-Al_2_O_3_ (0001) interface were investigated in more detail using STS (Fig. 6). In particular, measurements were made along a line at the boundary between G-II, which is nearly-free graphene, and G-I, which has a stronger interaction with sapphire, as indicated by the superstructure formed. The conductance and differential conductance spectra started on G-II, crossed a transition region, and reached G-I (see also Fig. 7a). They were performed at each point of the line (15 points) with different set point currents of 0.11 nA, 0.22 nA, 0.35 nA, 0.51 nA, and 0.64 nA defining different STM tip-surface distances. The full set of differential conductivity spectra can be found in Supporting Information (Fig. S4), while in Fig. 6, a selected set of normalized (d*I*/d*V*)/(*I*/*V*) spectra is plotted to show the main features. The STS curves in Fig. 6 are plotted at three different points, on G-II, in the transition region between G-II and G-I, and on G-I, for three representative set point currents of 0.22 nA, 0.35 nA, and 0.51 nA.

The local density of states (LDOS) in graphene is very sensitive to even small environmental pertubations such as electrostatic coupling to the underlying substrate, the effects of ripples and defects, or simply an approaching tip^59,62^. All these effects an lead to peculiarities in the bias dependence of the differential conductance, which is directly related to the local DOS. In the (d*I*/d*V*)/(*I*/*V*) curves obtained for G-II (Fig. 6a), two main features appear for the largest tip-sample distance (*I*_set_ = 0.22 nA) at (− 0.52 ± 0.03) V and (+ 0.36 ± 0.04) V, respectively. They correspond to tip-graphene interactions that increase in intensity with rising set point current, i. e., with decreasing tip-sample distance^63^. In Fig. 6d the minimum of the V-shaped Dirac point identified in the differential conductance of G-II can be discerned at *V*_DII_ = (15 ± 7) mV (*I*_set_ = 0.22 nA) indicating weak *p*-doping, which is consistent with the results of our van der Pauw measurements. However, this Dirac point shift strongly depends on the tip material, in particular the difference in work function between the tip material and the graphene, the tip geometry, and the distance between the tip and the sample^62–64^. As the tip approaches the sample, we observe a shift of the Dirac point and the whole STS spectrum to negative values, Δ*V*_DII_ = (-155 ± 13) mV (indicated by the vertical black line in Fig. 6a). This behaviour is consistent with data from the literature^63^.

The two shoulders flanking the Dirac point with a separation of about (134 ± 24) mV, observed for G-II in Figs. 6a,d and 7c, are attributed to electron–phonon (e–ph) interactions analogous to studies of graphene on SiO_2_ or on graphite^61,65^. The coupling of tunneling electrons with substrate inherent phonons, here presumably the out-of-plane acoustic phonon of graphene with an energy of about 65 meV^65^ leads to features in the LDOS at *E*_F_ ± 65 meV that explain our observations. e–ph coupling is characteristic for free-standing graphene and therefore suggests that at most a small interaction of SLG and sapphire may be present in G-II^61,64,65^. It should be noted that as the distance between the tip and the samples decreases, the pertubations of the LDOS become more pronounced, thus affecting the shape as well as the observation of the Dirac cone.

The normalized differential conductance data measured on G-I, (Fig. 6c) show significantly different features compared to G-II. The main features occur around (− 0.75 ± 0.02) V and (+ 0.85 ± 0.03) V and are assigned to interactions of the π-band of SLG with defect states of α-Al_2_O_3_ (0001). These defect states are reported to show a separation of about 1.5 eV and vary locally as expected for non-uniform surfaces^66^. Thus, the main features in the differential conductance spectra on G-I and G-II are due to different causes: SLG π-band/α-Al_2_O_3_ defect state and SLG/tip interaction, respectively. This is also reflected in the changed distance between the main features of 1.60 V on G-I and only 0.88 V on G-II (Fig. 7b) indicating clearly different interactions of SLG with the sapphire substrate in the two regions. As the W tip approaches G-I (*I*_set_ = 0.22 nA, 0.35 nA, 0.51 nA), a shift of the Dirac point of only Δ*V*_DI_ = (− 34 ± 14) mV (to negative voltages) is observed, which is significantly smaller compared to that on G-II. This is a consequence of the enhanced coupling of SLG to the α-Al_2_O_3_ (0001) substrate. It should be noted that at *I*_set_ = 0.22 nA the Dirac point is at *V*_DI_ = (16 ± 7) mV, indicating weak *p*-type doping, while at *I*_set_ = 0.51 nA the value *V*_DI_ = (− 18 ± 9) mV is the result of interactions of the G-I layer with the W-tip and the α-Al_2_O_3_ (0001) substrate. The Dirac points of the G-I and G-II regimes for large tip/substrate distances both show comparable slight *p*-doping.

The electrostatic binding of SLG on the enter of the terraces of α-Al_2_O_3_ (0001) in an epitaxial manner causes a periodic superstructure accompanied by a pronounced influence on the electronic structure of G-I. The periodic interlayer coupling leads to a breaking of the graphene sublattice symmetry and the twist angle between the SLG and α-Al_2_O_3_ (0001) discussed above probably leads to Van Hove singularities (VHS), i. e., maxima, in the LDOS of graphene^61^. These appear in our STS measurements on G-I as peaks at both sides of the Dirac point (Fig. 6c) with a separation of about ΔVHS = (301 ± 34) mV, confirming the interaction of SLG with the α-Al_2_O_3_ (0001) surface, since VHS as well as all other pertubations of graphene DOS with respect to the substrate can be observed only in the case of interlayer coupling (further analysis in Supporting Information).

The (d*I*/d*V*)/(*I*/*V*) curves depicted in Fig. 6b were obtained in the transition area and show features of both G-II and G-I with varying intensity. Interestingly, the STS curve measured at *I*_set_ = 0.35 nA shows a band gap opening at the Dirac point of (87 ± 5) meV (Fig. 6b,e; mean value over 6 measurements: (73 ± 3) meV), which is observed as a result of the interlayer interaction between SLG and the α-Al_2_O_3_ (0001) surface^27,33,67^. Such band gap openings can be as large as 90 meV or 260 meV, as reported for SiO_2_ or SiC substrates, respectively^68,69^. However, here the band gap is only visible at certain current set points used for STS and disappears at larger and smaller values^68^. We observe this dependence on the current set point, and, in addition, a local dependence. The band gap opening is best resolved at the last measurement point on G-I, where the periodic modulation of the π-band ends but the sublattice symmetry is still broken. As a result, the intensity of the VHS decreases and the interference with the remaining band gap is smaller. The magnitude of the band gap opening determined in this work is rather small and indicates a minor interaction between G-I and α-Al_2_O_3_ (0001). However, it agrees well with the value of 84 meV derived from advanced first-principle calculations performed by Huang et al. on the graphene/α-Al_2_O_3_ (0001) system^33^. Based on their calculations, the authors proposed two structures with clean interfaces. These are graphene on Al-terminated α-Al_2_O_3_ (0001) and graphene on fully hydroxylated α-Al_2_O_3_ (0001) with an open band gap of about 182 meV and 84 meV, respectively. On the other hand, the calculations of Huang et al. predict a strong interaction between an O-terminated sapphire surface and the graphene layer, leading to gap states resulting from hybridization between graphene and the oxygen orbitals of the surface^33^. Strong Al–O–C bonds, as described in Refs.^26,27,^ can be excluded for our system.

The changes in the local electronic properties of the graphene that occur along the line from one SLG region to the other are summarized in Fig. 7. Shown here are the peak positions along the line crossing the boundary between G-II and G-I determined from the STS analysis at 15 measurement points. Fig. 7b shows and compares the main characteristics of the (d*I*/d*V*)/(*I*/*V*) curves for the 0.22 nA and 0.35 nA set point currents, indicated by red and blue colours, respectively. The abrupt change of the main peak positions in the transition region between G-II and G-I can be clearly observed. In addition, the clear Dirac point dependence of G-II and G-I on the tip-to-surface distance is evident. Another interesting feature, best inferred from Fig. 7c, is the alternating positions of the VHS peaks (regime G-I) and peaks in G_II resulting from e–ph coupling. As mentioned above, e–ph coupling can only be observed in a (nearly) decoupled graphene layer, while VHS results from a coupling of twisted layers. Thus, the origin of the peaks around *V*_D_ in G-I and G-II is clearly different and directly supports the assumption that G-I corresponds to coupled graphene, while G-II is almost free-standing.

The results of the comprehensive analysis of the SLG/α-Al_2_O_3_ (0001) system obtained from a scalable CVD process by means of chemical surface characterization and quantitative analysis of the local topography and electronic structure in comparison with studies of graphene on graphite and SiO_2_ and structure simulations derived from first principle calculations^33,70^ allow us to unambiguously identify the interlayer coupling of the SLG/α-Al_2_O_3_ (0001) interface. Based on the results of XPS and (d*I*/d*V*)/(*I*/*V*) analysis, we can exclude the O-terminated α-Al_2_O_3_ (0001) surface and strong Al-O-C bonds for our system. A more detailed comparison identifies the SLG/hydroxylated α-Al_2_O_3_ (0001) as the most likely interface. This is also supported by the flat topography observed for the SLG on the sapphire terraces and by the experimental evidence of a band gap opening in the SLG of about (73 ± 3) meV at the Dirac point. This is very close to the value of about 84 meV calculated for the interface of graphene and the hydroxylated α-Al_2_O_3_ (0001) surface^33^.

**Figure 8:**
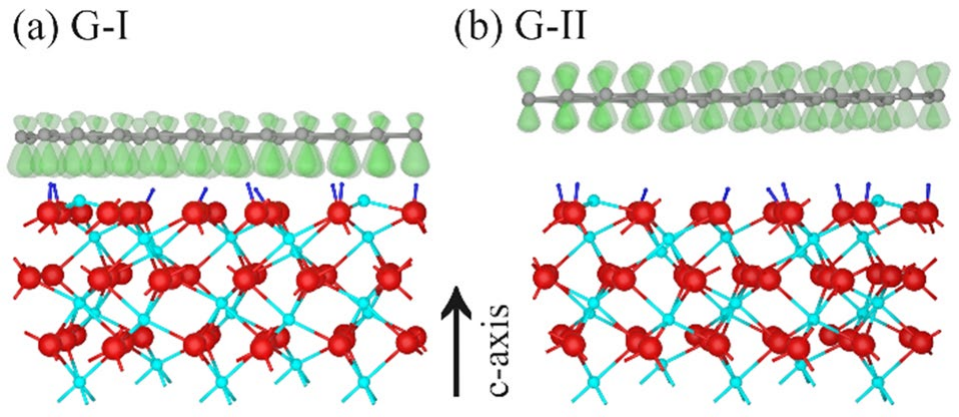
Illustration of the SLG/α-Al_2_O_3_ (0001) interface in the G-I and G-II regimes (Blender Version 2.93.1; http://www.blender.org). The atomic structure of α-Al_2_O_3_ in the c-plane is taken from Jain et al.^72^ (Al turquoise, O red) and a proposed hydroxyl-termination added (H blue) in agreement with Ref.^33^. SLG is shown in grey, the π-electron system in green. (**a**) Interaction of hydroxyl-terminated sapphire with SLG (region G-I). (**b**) ‘nearly-free-standing’ SLG (region G-II).

In summary, the schematic drawings in Fig. 8 present the most reasonable SLG/hydroxylated α-Al_2_O_3_ (0001) structures for the two regimes in agreement with the chemical and topographic results described so far. The partial hydroxy -termination of the sapphire surface derived from XPS analysis is included in the schematics. The SLG regime G-I shown in Fig. 8a is characterized by the interaction of the delocalized graphene-π-electron system with the top sapphire layer, leading to weak hydrogen bonds of the O–H…π-electron system^33,71^. In the G-II region near the step edges, the interlayer spacing is further increased, resulting in nearly free-standing SLG, as shown in Fig. 8b. In summary, these differences in the interlayer coupling at the SLG/α-Al_2_O_3_ (0001) interface in the G-I and G-II regions explain our experimental topographic and electronic observations. The basic model shown in Fig. 8 serves as a starting point for discussing the effects of interlayer coupling on the electronic properties of devices based on graphene and, moreover, stacked 2D-materials.

## Conclusions

In summary, we have investigated local correlations between morphological, topographical and electronic properties of SLG deposited on H_2_-etched α-Al_2_O_3_ (0001) applying various surface sensitive methods such as SEM, AFM, c-AFM in combination with XPS and Raman spectroscopy. The homogeneous wafer-size SLG which originates from a commercial CVD process, shows inhomogeneity at the local level at intermediate defect concentration. In addition, we have identified two regions of our SLG films with significantly different SLG/α-Al_2_O_3_ (0001) interfacial interactions. Characteristically, these regions are located either on sapphire terraces or along step edges. Based on an atomically resolved topographic and electronic characterisation using STM/STS methods, weak but distinct interfacial interactions were found on the sapphire terraces, which can be attributed to weak hydrogen bonds between the hydroxyl terminated sapphire and the SLG. These to moiré structures  formed by a twist angle between the hexagonal α-Al_2_O_3_ (0001) and the hexagonal graphene structure. A band gap opening in the SLG of about (73 ± 3) meV at the Dirac point, which is a consequence of charge injection into the graphene layer, could be experimentally detected. The absolute value is in good agreement with a prediction from first-principle calculations. In contrast, SLG near the step edges is considered to be almost free-standing. This situation can be described as conductive paths formed by the free-standing SLG along the sapphire step edges and the SLG on the sapphire terraces, which is less conductive by a factor of 4. The weak interfacial interaction between SLG and H_2_-etched sapphire allows for high charge carrier mobility leading to high conductivity of SLG/α-Al_2_O_3_ (0001). We believe that these results contribute to further understanding of the SLG/α-Al_2_O_3_ (0001) interface and are of particular interest for future device concepts based on graphene as conducting electrode layer with epitaxial relationship to the supporting insulating substrate.

## Supplementary Information

Supplementary Information.

## Data Availability

The datasets used and/or analyzed during the current study are available from the corresponding author on reasonable request.
