# Supplementary material for: Atomically resolved electronic properties in single layer graphene on α-Al2O3 (0001) by chemical vapor deposition
Source: Sci Rep. 2022 Nov 5;12:18743. doi: 10.1038/s41598-022-22889-4 (PMC9637179; doi:10.1038/s41598-022-22889-4)
Supplement: Supplementary file 1 — Supplementary Information. [file 41598_2022_22889_MOESM1_ESM.pdf]

Supplementary Information

Atomically resolved electronic properties in single layer graphene on α-Al_2O_3 (0001) by chemical vapor deposition

Henrik Wördenweber [1,6], Silvia Karthäuser [1,], Annika Grundmann [4], Zhaodong Wang [1,6], Stephan Aussen [1,6], Holger Kalisch [4], Andrei Vescan [4], Michael Heuken [4,5], Rainer Waser [1,2,3], and Susanne Hoffmann-Eifert [2,*]*

[1] Peter Grünberg Institute 7, Forschungszentrum Jülich GmbH and JARA-FIT, 52425 Jülich, Germany

[2] JARA-Institute Energy-Efficient Information Technology (Green IT & PGI-10), Forschungszentrum Jülich GmbH, 52425 Jülich, Germany

[3] Institute of Materials in Electrical Engineering and Information Technology II, RWTH Aachen University, 52074 Aachen, Germany

[4] Compound Semiconductor Technology, RWTH Aachen University, 52074 Aachen, Germany

[5] AIXTRON SE, 52134 Herzogenrath, Germany

[6] RWTH Aachen University, 52066 Aachen, Germany

Content

- Raman mapping and Raman spectroscopy
- X-ray Photoelectron Spectroscopy (XPS) survey scans of the pristine α-Al_2O_3 (0001) substrate, sapphire pre-baked in hydrogen at 1400 °C, and the SLG/sapphire sample as well as Al 2p, O1s, and C1s core level spectra of the differently treated substrates.
- Atomic Force Microscopy (AFM) image performed on SLG/α-Al_2O_3(0001) with multiple different sapphire steps
- Scanning Electron Microscopy (SEM) images of SLG/α-Al_2O_3(0001) in top view
- STM of the superstructure in regime G-I of SLG/α-Al_2O_3(0001) and analysis of the moiré structure
- STS full data set
- Van Hove singularities

Figure S1 (a) Raman mapping with marked positions indicating SLG under compressive strain

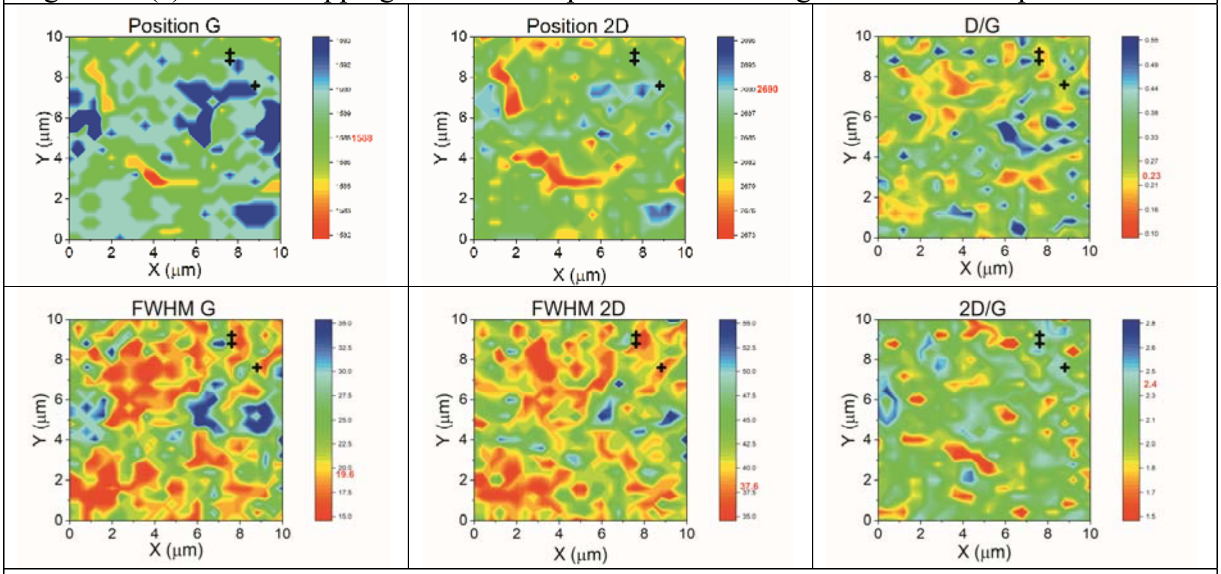

Figure S1 (b) Raman mapping with marked positions that indicate unstrained SLG

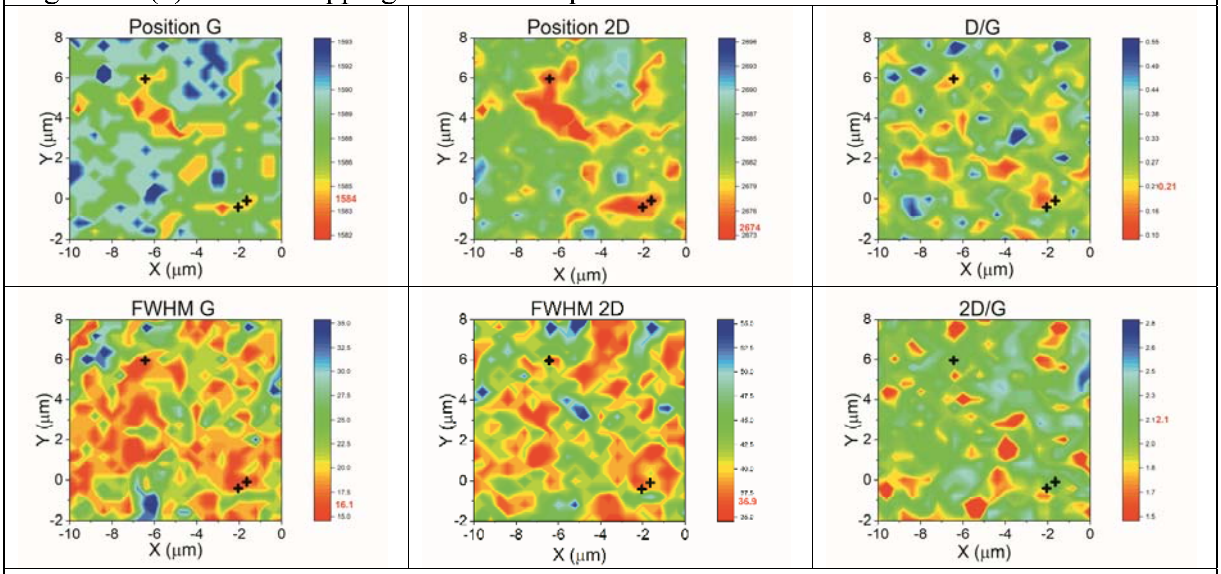

Figure S1 (c) Raman mapping with marked positions that indicate multilayer graphene

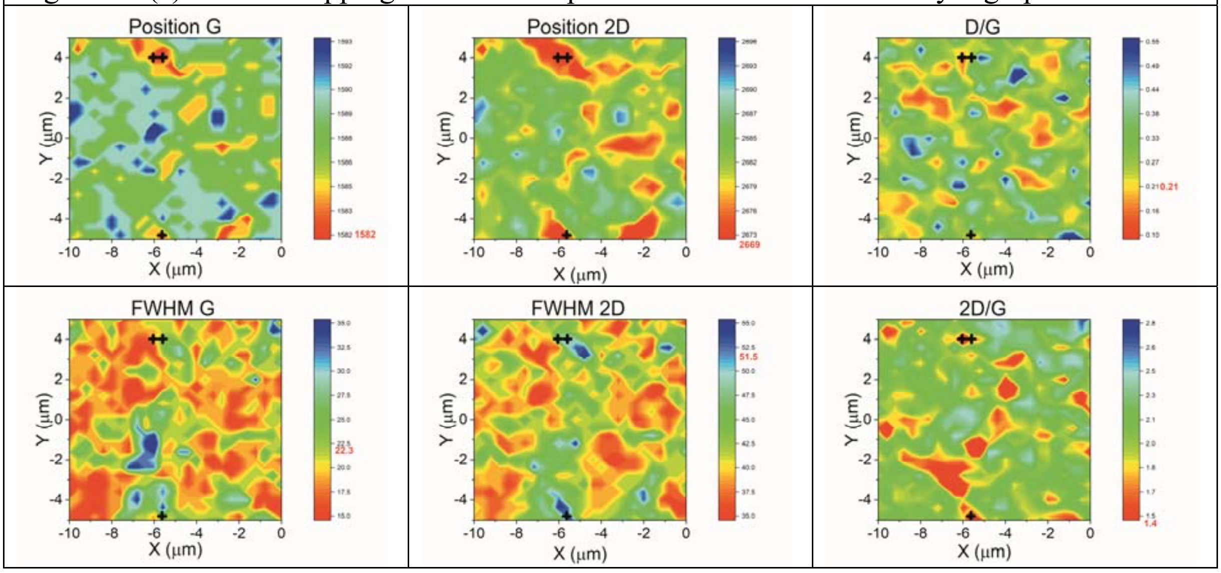

Figure S1 (d) Raman spectra of the positions marked in Figures S1 (a) to (c)		
SLG under compressive strain	Unstrained SLG	Multilayer graphene
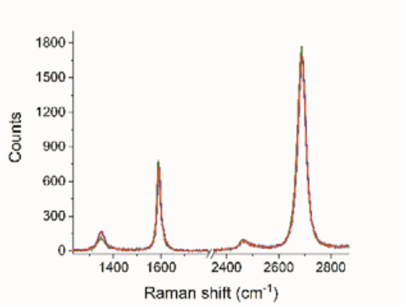	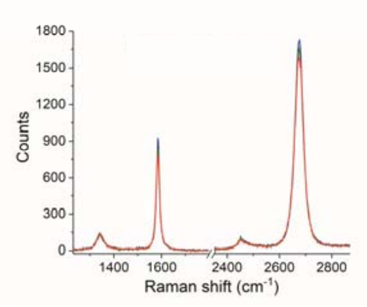	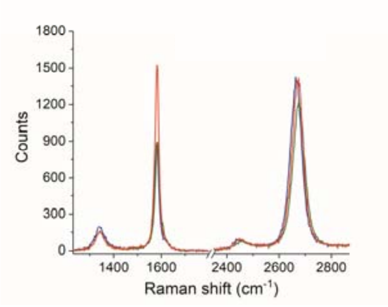

Figure S1. (a)-(c) Raman mappings performed on SLG/α-Al_2O_3 (0001) with areas of 10x10 μm^2 at a spot size of 0.5 μm in diameter and 0.4 μm step size. The markers indicated the positions where the different spectra shown in (d) are taken. The characteristic values are summarized in Table S1.

Table S1 Characteristic values of each spectrum shown in Figure S1 (d)

	Line	I_D/I_G	I_{2D}/I_G	$\Delta\omega_G$ [cm^{-1}]	$\Delta\omega_{2D}$ [cm^{-1}]	$FWHM_G$ [cm^{-1}]	$FWHM_{2D}$ [cm^{-1}]
SLG under compressive strain	blue	0.27	2.4	1589	2691	18.7	36.8
	green	0.18	2.4	1587	2688	21.4	39.1
	red	0.25	2.4	1589	2691	18.7	36.8
	Average	0.23±0.06	2.4±1	1588±1	2690±2	20±2	38±2
Unstrained SLG	blue	0.19	2.2	1584	2674	16.1	34.6
	green	0.21	2.1	1584	2674	16.1	36.9
	red	0.22	2.1	1584	2674	16.1	39.2
	Average	0.21±0.02	2.1±0.1	1584±1	2674±1	16±1	37±3
Multilayer graphene	blue	0.27	1.7	1582	2665	24.1	53.1
	green	0.22	1.5	1582	2672	24.1	53.0
	red	0.13	1.0	1582	2670	18.8	48.4
	Average	0.21±0.08	1.4±0.4	1582±1	2669±4	22±4	52±4

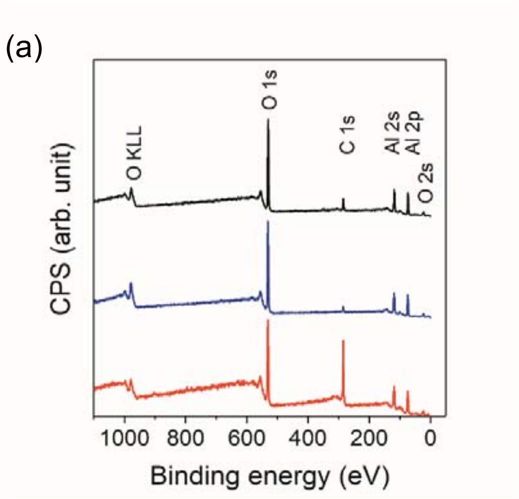

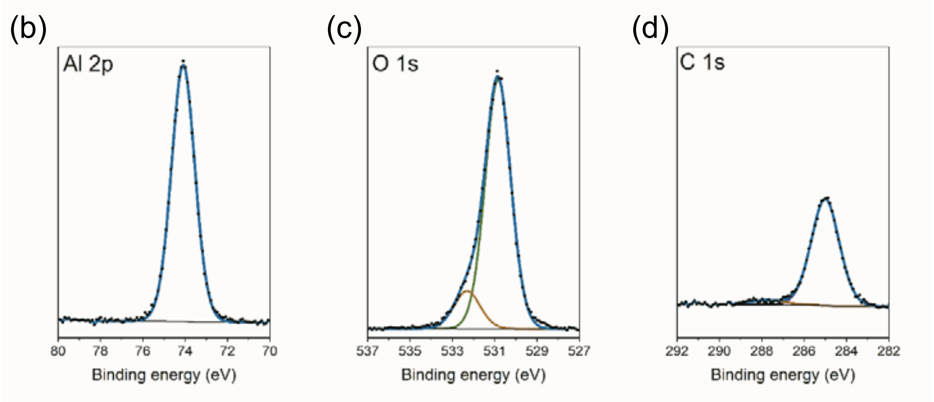

Core level	Al 2p		O 1s			C 1s	
Component	Al_2O_3	Al-OH	Al_2O_3	Al-OH	C=O	C sp^3	C=O
Binding energy [eV]	74.1	-	530.8	-	532.3	285.0	287.8

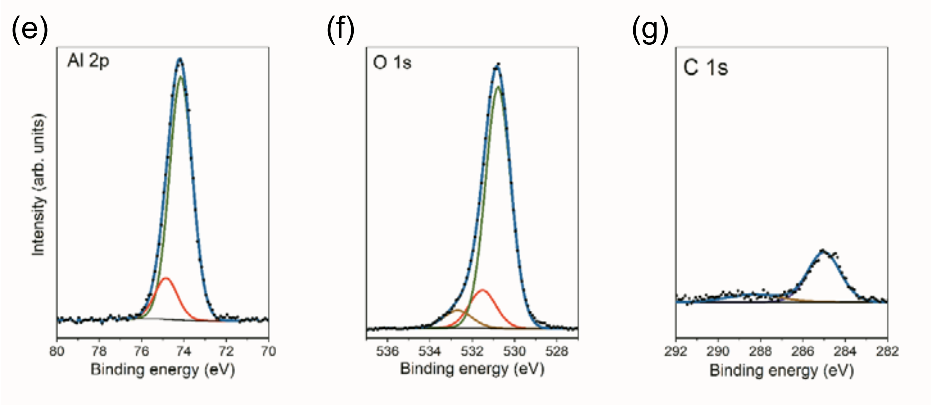

Core level	Al 2p		O 1s			C 1s	
Component	Al_2O_3	Al-OH	Al_2O_3	Al-OH	C=O	C sp^3	C=O
Binding energy [eV]	74.1	74.8	530.8	531.5	532.7	285.0	288.3

Figure S2. (a) XPS survey scans of the pristine α-Al_2O_3 (0001) (black), the H_2-pre-baked sapphire surface (blue), and the SLG/sapphire samples (red). (b) – (f) Core level spectra for a take-off angle of 45° and a pass energy of 11.75 eV: (b)-(d) pristine sapphire, (e)-(f) H_2-pre-baked sapphire. The black dots show the raw data, the steel blue lines show the envelopes of the fitted components, and the black lines show the subtracted Shirley backgrounds. The following components are used for fitting: Al-OH in red, Al_2O_3 in green, C=O in brown, C sp^3 in grey.

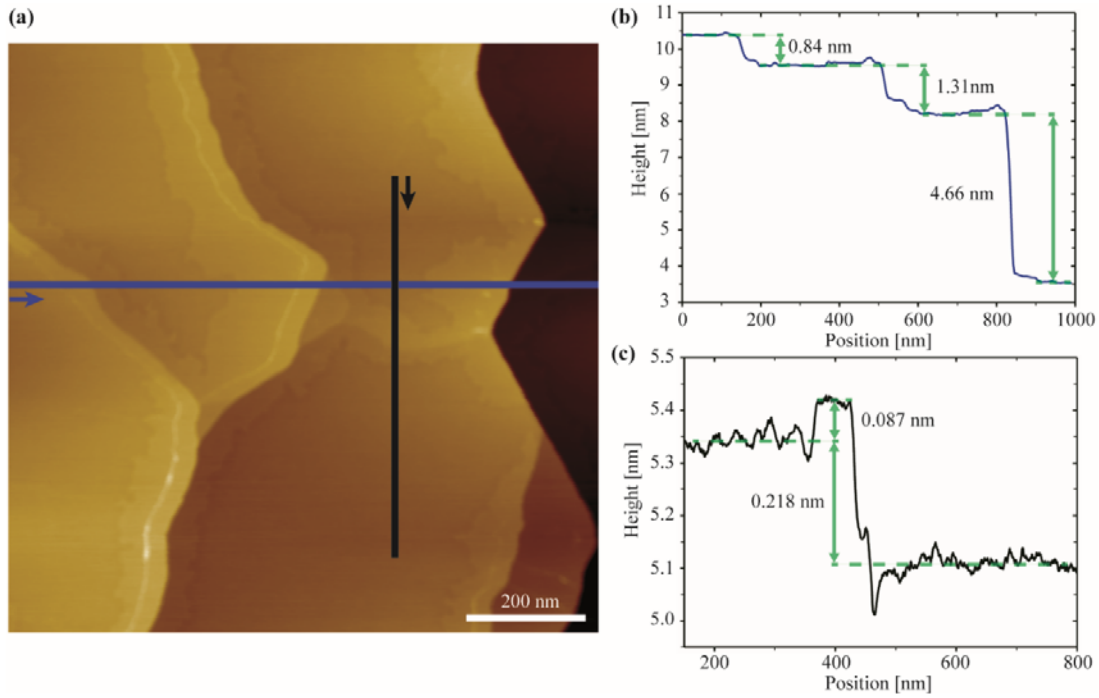

Figure S3. Atomic Force Microscopy (AFM) image performed on SLG/α-Al_2O_3 (0001) with multiple different sapphire steps

Figure S3 shows an Atomic Force Microscopy (AFM) image performed on single layer graphene on sapphire, SLG/α-Al_2O_3 (0001). Figure S3 (a) corresponds to the AFM images presented in the main paper (cf. Figure 3 (a), 3 (b)). Additionally, two line profiles are given in (b) and (c). These line profiles reveal the presence of the different sapphire step edges. In (b) the line profile throughout the whole images shows multiple different sapphire steps. The three marked steps have heights of 0.84 nm, 1.31 nm, and 4.66 nm, which correspond to a step over four oxygen layers, a unit cell step, and a combination of three unit cells and three oxygen layers, respectively. In (c) a line profile across a single oxygen layer step of 0.218 nm is shown. A graphene region change from G-II to G-I, like discussed in the main paper, is also present here (height of 0.087 nm).

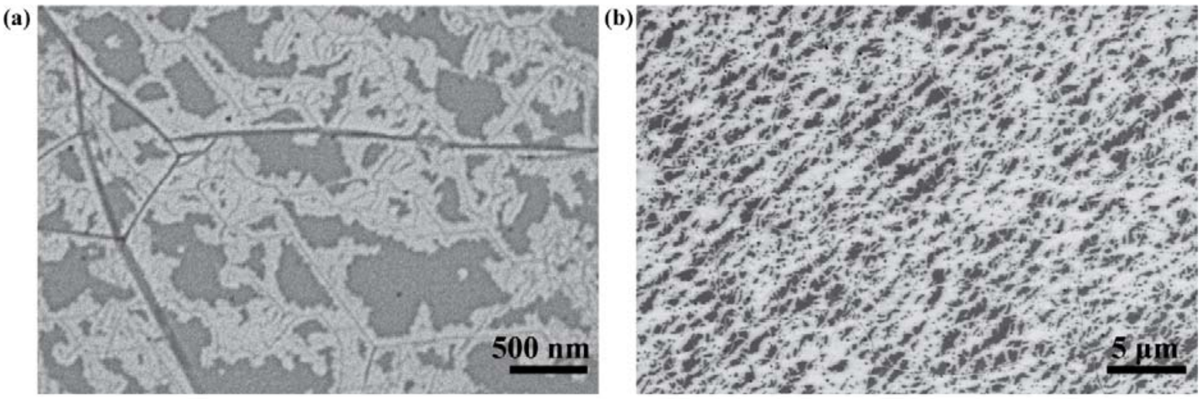

Figure S4. Scanning Electron Microscopy (SEM) images of SLG/α-Al_2O_3 (0001) in top view

Figure S4 shows Scanning Electron Microscopy (SEM) images performed on single layer graphene on sapphire, SLG/α-Al_2O_3 (0001), samples. Image (a) presents a slightly larger version of the image shown in the paper (cf. Figure 3 (e)). The two different regions of graphene (G-I and G-II) and the sapphire step edges are visible as the bright and dark contrast areas and as the small meandering lines, respectively. Figure S4 (a) demonstrates the distribution of the two regions G-I (dark) and G-II (bright), with the weak electrostatically bonded graphene (G-I) located on the sapphire terraces while the delaminated graphene (G-II) is present close to sapphire step edges. In (b) a representative overview SEM image of the SLG/α-Al_2O_3 (0001) sample is presented. This large scale image demonstrates an even distribution of the different graphene regions, G-I and G-II. These images are taken arbitrarily throughout the whole wafer and enable a quick overview confirming this pattern.

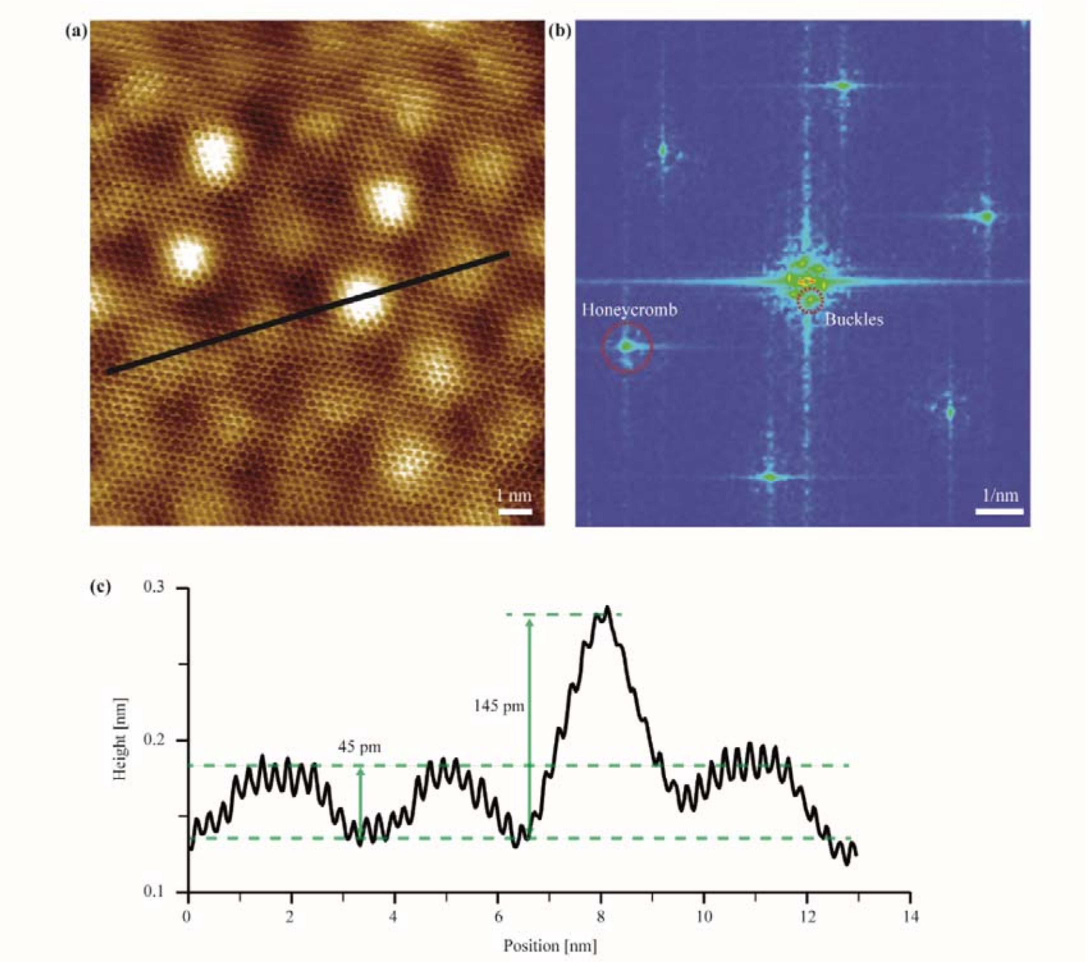

Figure S5. STM of the superstructure in regime G-I of SLG/α-Al_2O_3 (0001)

Details of the superstructure determined in region G-I by Scanning Tunnelling Microscopy (STM) are given in Figure S5. (a) STM image of this moiré structure resulting from a twist angle between the hexagonal α-Al_2O_3 (0001) and the hexagonal graphene structure, both with not too different lattice constants (in-plane lattice constant of c-plane sapphire = 0.476 nm [1], graphene lattice constant = 0.246 nm). This STM image was taken at V_{bias} = 2.3 V and I_{set} = 0.23 nA. (b) shows the Fast Fourier Transformation (FFT) of the STM image in (a). The two visible periodic structures, the graphene honeycomb structure and the buckle forming superstructure, are visible in this FFT image as outer hexagon and inner hexagon, respectively. The FFT reveals the periodicity of these structures resulting in (2.66 ± 0.03) nm for the moiré structure and (0.25 ± 0.01) nm for the graphene honeycombs, which is comparable to the expected graphene lattice at 4 K of 0.25 nm [2] and previously reported superstructures of graphene on sapphire [3]. Additionally, the rotational angle between the structures can be measured to be 26.7° ± 1°. A line profile of the STM image is marked in (a) and shown in (c), with the buckles clearly visible. Most of these buckles have a height of approx. 45 pm, with some seemingly randomly distributed single buckles exhibiting heights of up to 110-150 pm.

Analysis of the moiré structure

The moiré pattern depicted in Figure 5 (a) for the system SLG/α-Al_2O_3 (0001) shows a translational symmetry with a period of L = (2.66 ± 0.03) nm. One condition for a commensurate superstructure is that L corresponds to a multiple of the unit cell of graphene, a_o = 0.246 nm,

$$L = a_0 \cdot (3i^2 + 3i + 1)^{\frac{1}{2}} \quad \text{with i = integer [4].} \qquad (1)$$

Applying equation (1) we find i = 5.74 for a_o = 0.246 nm, but i ≈ 6 for a = (0.476 nm)/2, that is half of the in-plane lattice constant of c-plane sapphire. It seems reasonable that the buckling of graphene results from the adoption of the hexagonal graphene lattice to the sapphire substrate.

The strain resulting from this contraction is $\delta = (a - a_o) / a_o = -0.032$.

Using the relationship between the rotation angle in K-space (φ) and θ, denoting the twist angle between the hexagonal moiré pattern and the hexagonal graphene structure in real space [5],

$$\varphi = (30° - (\frac{\theta}{2})) \qquad (2)$$

and employing φ = 26.7° ± 1° from FFT we deduce θ = 6.6° ± 2°.

However, a more precise value for the twist angle can be deduced from the period of the moiré pattern [5]:

$$L = \frac{a_0}{2 \cdot \sin(\frac{\Theta}{2})} \qquad (3)$$

From (3) and the use of our measured value for L a real-space twist angle θ = 5.1° results which we use for the estimations of ΔVHS.

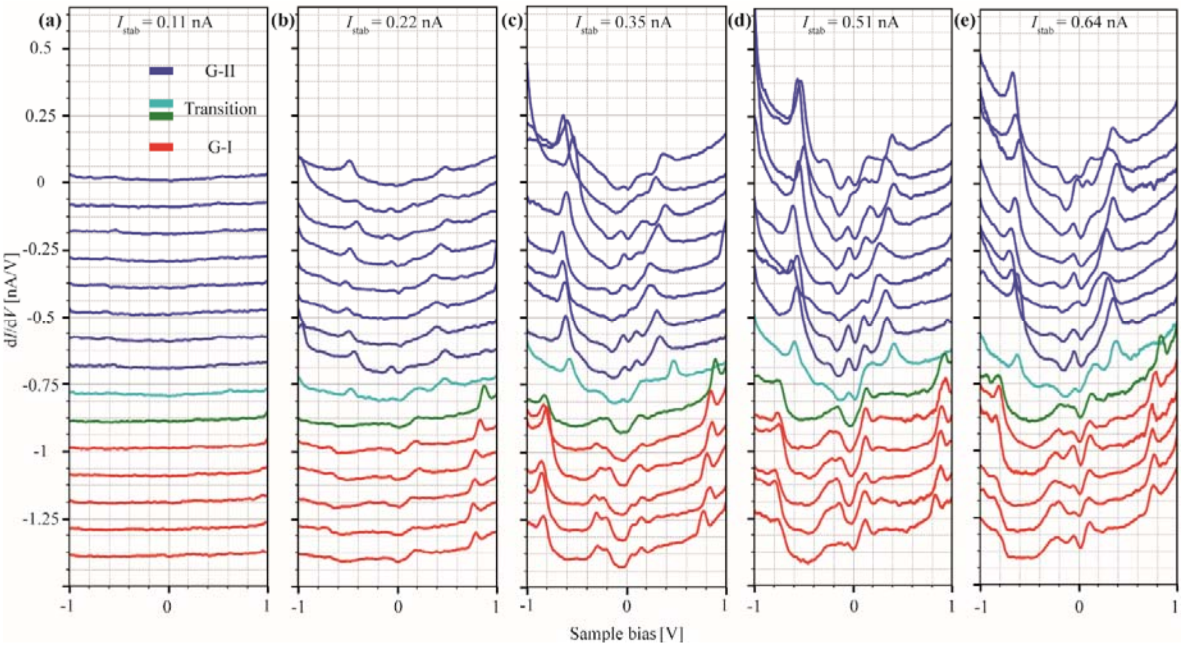

Figure S6. STS full data set

In Figure S6 the complete Scanning Tunneling Spectroscopy (STS) data-set used in the analyses is provided. STS measurements were performed on positions along a line crossing the border between G-II and G-I using multiple set-point currents (I_{set}). The position of the STS measurements are shown in Figure 7 (a) of the main paper. Here the STS curves for all positions are plotted in one diagram for a given current set-point. The first 8 measurements located on the G-II area are plotted in blue, followed by the two transition area measurements in cyan and green, and ends with the 5 positions on the G-I area in red. For clarity, succeeding STS curves are shifted downwards by 0.1 nA/V each. The measurement sets for the different I_{set} (0.11 nA, 0.22 nA, 0.35 nA, 0.51 nA, 0.64 nA), corresponding to the approach of the tungsten tip towards the surface, are plotted in (a), (b), (c), (d), (e), respectively.

Van Hove singularities (VHS)

The twist-induced moiré pattern in the system SLG/α-Al_2O_3 (0001) has also an effect on the electronic band structure of SLG. Due to the periodic modulation of the interface interactions between SLG and the surface states of the H_2-etched α-Al_2O_3 (0001) Van Hove singularities, that are divergences in the DOS of SLG, are induced. They appear on both sides of the Dirac point and their separation (ΔVHS) depends on the twist angle between the moiré pattern and SLG. In region G-I we obtained ΔVHS = (301 ± 34) meV around V_{DI} = (16 ± 7) mV at I_{set} = 0.22 nA.

A first attempt to make an estimation of ΔVHS is based on [6]. Here, ΔVHS is given as a function of the Fermi velocity, v_F^o, the twist angle, θ, and the nearest neighbor hopping energy perpendicular to the graphene layer (also denoted as transfer integral), $t_\perp$ = 0.28 eV for SLG:

$$\Delta VHS \approx \hbar \cdot v_F{}^{\circ} \cdot \Delta K - 2 \cdot t_{\perp}^{\Theta} \tag{4}$$

with $$\Delta K = \left(\frac{4\pi}{3} \cdot \sqrt{3} \cdot a_0\right) \cdot \sin\left(\frac{\Theta}{2}\right) \tag{5}$$

Applying a_o = 0.246 nm, θ = 5.1°, $v_F{}^{\circ} = 1\ 10^6$ m/s for SLG, and $t_{\perp}{}^{\theta} \approx 0.4\ t_{\perp}$, like given in [6] leads to ΔVHS(calc.) = 677 meV, which would be an extrapolation of the curve ΔVHS = f(θ) in [6] which is only covering the range: 1° < θ < 3.5°. However, this method of calculating ΔVHS can not be applied in our case.

So far, this approach presented by [6] does not comprise that the Fermi velocity is dependent on the twist angle [6], is inversely proportional to the dielectric constant of the substrate (ε = 11.5 parallel to c-axis of sapphire) [7], and decreases with increasing carrier densities [7]. In contrast to [6], who discussed twisted graphene layers on graphite with an interlayer spacing around 0.34 nm, we have to assume considerably shorter interlayer distances between SLG and H_2-etched α-Al_2O_3 (0001) in the range of 0.231 nm to 0.318 nm (due to a rippled surface [8]). A substantially increased interface coupling leads to a decrease in electron velocity, like observed in [9]. This will directly affect the transfer integral, $t_{\perp}$, which will be increased due to the increased coupling between the π-bonds perpendicular to graphene with the dangling bonds originating from H_2-etched α-Al_2O_3 (0001).

The best choice would be to probe the Fermi velocity in our system by terahertz spectroscopy [7], Landau level spectroscopy [5], or an interesting method introduced recently, d*I*(*V*)/dz-spectroscopy [10]. While the first two methods are not available in our case, the latter one is probed so far for SLG deposited on a substrate without electronic features in the DOS around V_D leaving the intrinsic band structure of graphene intact. This is not given in our case. Hence, for the next attempt to estimate ΔVHS we use v_F^{Θ} in equation (4) instead of $v_F{}^{\circ}$ and make an educated guess based on the dependencies of v_F^{Θ} on θ, ε, and n (charge density = n = $2.22 \cdot 10^{12}$ cm^{-2} from our Hall measurements) given in literature [6,7]. We propose for our twisted, interface coupled system SLG/H_2-etched α-Al_2O_3 (0001):

$$v_F^{\Theta} = v_F(free\ SGL) \cdot F_1 \cdot\ F_2 \cdot\ F_3 = 0.77 \cdot 10^6\ m/s \tag{6}$$

with $v_F(free\ SGL) = v_F^0 = 1.1 \cdot 10^6\ m/s$ [11], F_1 = 0.9 factor comprising the twist angle dependence (5°) [6], F_2 = 0.85 factor including the dependence on the dielectric constant of the substrate [7], and F_3 = 0.92 with respect to the dependence on the charge density [7]. This value is in the range of other reported Fermi velocities [6].

Following [6] it is possible to estimate the coupling strength using

$$\frac{v_F^{\Theta}}{v_F^0} = 1 - 9 \cdot \left(\frac{t_{\perp}^{\Theta}}{\hbar \cdot v_F^0 \cdot \Delta K}\right)^2 . \tag{7}$$

With $v_F^{\Theta} = 0.77 \cdot 10^6\ m/s$ a value of $t_{\perp}{}^{\theta}$ = 0.18 eV is obtained. Using Formula (4) we can estimate ΔVHS(calc.) = 337 meV, which is consistent with our measured value.

REFERENCES

[1] Cuccureddu, F., Murphy, S., Shvets, I. V., Porcu, M., Zandbergen, H. W., Sidorov, N. S., Bozhko, S. I. Surface morphology of c-plane sapphire (alpha-alumina) produced by high temperature anneal. *Surf. Sci.* **2010**, *604*, 1294-1299.

[2] Magnin, Y., Foerster, G. D., Rabilloud, F., Calvo, F., Zappelli, A. & Bichara, C., Thermal expansion of free-standing graphene: benchmarking semi-empirical potentials. *J. Phys. Condens. Mat.* **2014**, *26*, 185401/1-10.

[3] Mishra, N., Forti, S., Fabbri, F., Martini, L., McAleese, C., Conran, B. R., Whelan, P. R., Shivayogimath, A., Jessen, B. S., Buss, L., Falta, J., Aliaj, I., Roddaro, S., Flege, J. I., Boggild, P., Teo, K. B. K., Coletti, C. Wafer-scale synthesis of graphene on sapphire: Toward fab-compatible graphene. *Small* **2019**, *15*, 1904906/1-8.

[4] Li, G., Luican, A., Lopes dos Santos, J. M. B., Castro Neto, A. H., Reina, A., Kong, J., Andrei, E. Y. Observation of Van Hove singularities in twisted graphene layers. *Nat. Phys.* **2010**, *6*, 109-113.

[5] Luican, A., Li, G., Reina, A., Kong, J., Nair, R. R., Novoselov, K. S., Geim, K. S., Andrei, E. Y. Single-Layer Behavior and Ist Breakdown in Twisted Graphene Layers. *Phys. Rev. Lett.* **2011**, *106*, 126802.

[6] Andrei, E. Y., Li, G., Du, X. Electronic properties of graphene: a perspective from scanning tunneling microscopy and magnetotransport. *Rep. Prog. Phys.* **2012**, *75*, 56501/1-47.

[7] Whelan, P. R., Shen, Q., Zhou, B., et al. Fermi velocity renormalization in graphene probed by terahertz time-domain spectroscopy. *2D Mater.* **2020**, *7*, 035009/1-11.

[8] Huang, B., Xu, Q., Wei, S. H. Theoretical study of corundum as an ideal gate dielectric material for graphene transistors. *Phys. Rev. B* **2011**, *84*, 155406/1-5.

[9] Wang, Z., Hao, Z., Yu, Y., et at. Fermi Velocity Reduction of Dirac fermions around the Brillouin Zone Center in In_2Se_3-Bilayer Graphene Heterostructures. *Adv. Mater.* **2021**, *33*, 2007503.

[10] Jiao, Z., Zandvliet, H. J. W. Determination of the Fermi velocity of graphene on MoS_2 using dual mode scanning tunneling spectroscopy. *Appl. Phys. Lett.* **2021**, *118*, 163103/1-5.

[11] Deacon, R. S., Chuang, K.-C., Nicholas, R. J., Novoselov, K. S., Geim, A. K. Cyclotron resonance study of the electron and hole velocity in graphene monolayers. *Phys. Rev. B* **2007**, *76*, 081406.
